# Cyberbullying victimization and perceived academic stress among vocational college students: the mediating roles of anxiety, insomnia, and self-control

**DOI:** 10.3389/fpsyg.2026.1865591

**Published:** 2026-08-26

**Authors:** Qingqin Zhu, Qiyan Feng, Qinghui Xu, Kejia Zhong, Hanfeng Chen

**Affiliations:** 1 School of Creative Studies, Yiwu Industrial and Commercial College, Jinhua, China; 2 College of Digital Media, Chongqing Technology and Business Institute, Chongqing, China; 3 School of Humanities and Tourism, Yiwu Industrial and Commercial College, Jinhua, China; 4 School of Computer Science and Technology, East China Normal University, Shanghai, China

**Keywords:** anxiety, cyberbullying victimization, insomnia, perceived academic stress, self-control, vocational college students, academic adjustment, vocational education

## Abstract

**Introduction:**

Cyberbullying victimization has become an important risk experience in students' digital lives, yet its association with education-specific outcomes, such as perceived academic stress, remains underexplored, especially among vocational college students. This study examined the association between cyberbullying victimization and perceived academic stress, with particular attention to theoretically informed statistical indirect pathways involving anxiety, insomnia, and self-control.

**Methods:**

Data were collected from vocational college students in Chongqing and Zhejiang, China. After data screening, 378 valid responses were retained. Participants completed standardized and research-version measures of cyberbullying victimization, anxiety, insomnia, self-control, and perceived academic stress. Analyses included descriptive statistics, Pearson correlations, confirmatory factor analysis, structural equation modeling, and bias-corrected bootstrap analysis with 5,000 resamples using SPSS 27.0.1 and AMOS 24.

**Results:**

Cyberbullying victimization was positively associated with anxiety, insomnia, and perceived academic stress, and negatively associated with self-control. Both the five-factor measurement model and the structural model showed good fit to the data. In the structural model, cyberbullying victimization was significantly positively associated with anxiety and perceived academic stress and negatively associated with self-control. Anxiety was positively associated with insomnia, insomnia was positively associated with perceived academic stress, and self-control was negatively associated with perceived academic stress. Bootstrap analyses supported significant total, direct, and indirect associations, indicating a statistical partial mediation pattern.

**Discussion:**

Cyberbullying victimization was associated with vocational college students' perceived academic stress both directly and through statistically supported indirect pathways involving anxiety–insomnia and self-control. These findings extend cyberbullying victimization research into the educational domain and provide preliminary implications for student identification, counseling, and support in vocational colleges. Given the cross-sectional self-report design, the observed pathways should be interpreted as theoretically grounded statistical associations rather than confirmed temporal or causal mechanisms.

## 1 Introduction

As digital media become deeply embedded in students' academic and social lives, cyberbullying victimization has emerged as a salient risk experience in higher education. Recent review evidence indicates that cyberbullying and cyberstalking victimization are not uncommon among university students and are frequently accompanied by emotional distress, interpersonal difficulties, and diminished wellbeing (Bussu et al., 2025). Within Chinese college populations, cybervictimization is increasingly conceptualized as an ongoing online interpersonal stressor rather than an isolated adverse event, suggesting that its influence may extend into subsequent psychological and behavioral adjustment processes (Luo et al., 2023).

A growing body of research has linked cyberbullying victimization to a range of maladaptive outcomes. Studies in adolescent and college samples suggest that cybervictimization is associated with emotional symptoms, stress-related responses, and impaired sleep, indicating that its consequences may extend beyond online interaction itself and spill over into everyday functioning (Fang et al., 2026; Staude-Müller et al., 2012; Kwon et al., 2020). However, the literature has focused more often on emotional symptoms, cyberbullying behavior, or sleep quality as outcomes, while paying comparatively less attention to whether cybervictimization may also contribute to perceived academic stress, a more education-specific outcome.

Positioning perceived academic stress as the outcome is theoretically and practically meaningful. Academic stress has repeatedly been identified as one of the most salient stressors in college students and is significantly associated with poorer mental well-being (Barbayannis et al., 2022). This issue may be especially relevant in the present vocational-college context. In this study, the academic-demand context was defined to include conventional coursework as well as skills training, practicum tasks, and internship-related requirements. Under these conditions, if cyberbullying victimization is associated with a stronger subjective sense of burden regarding academic tasks, performance requirements, and time pressure, its consequences may extend beyond mental health to educational adjustment.

Among the potential mechanisms, anxiety and insomnia constitute a plausible emotional–sleep pathway. Recent evidence indicates that both traditional victimization and cyber victimization may impair sleep quality through cognitive and emotional processes, with anxiety functioning as a particularly important mechanism (Wang et al., 2025). In parallel, systematic review and meta-analytic evidence has shown that poor sleep, insomnia symptoms, and stress are closely intertwined in undergraduate populations (Gardani et al., 2022). Taken together, these findings support the possibility that cyberbullying victimization may first activate anxiety, then disrupt sleep, and eventually intensify students' perceived academic stress by undermining their capacity to cope effectively with academic demands.

In addition to the emotional–sleep pathway, self-control may represent a complementary self-regulatory pathway. Previous research among college students has linked self-control with learning engagement, goal-directed behavior, and academic functioning (Yang et al., 2024; Duckworth et al., 2019). Accordingly, the proposed mediation model integrates an emotional–sleep pathway involving anxiety and insomnia with a self-regulatory pathway involving self-control.

Against this background, the present study focused on Chinese vocational college students and examined the relationship between cyberbullying victimization and perceived academic stress. More specifically, it tested two potential mechanisms simultaneously: first, the serial mediating roles of anxiety and insomnia; and second, the mediating role of self-control. The expected contributions of the present study are threefold. First, it extends the outcome of cyberbullying victimization from general negative emotional responses to perceived academic stress in an educational context. Second, it integrates an emotional–sleep pathway and a self-regulatory pathway within a single model. Third, it pays particular attention to Chinese vocational college students, a population that has been comparatively underrepresented in recent cybervictimization research. In doing so, the study aims to provide educational-psychological evidence regarding how digital victimization experiences may spill over into students' academic adjustment.

## 2 Theoretical framework and hypothesis development

### 2.1 Cyberbullying victimization and perceived academic stress

Cyberbullying victimization can be understood as an ongoing form of digital interpersonal stress rather than a single adverse online event. Compared with offline conflicts, online victimization may involve potential anonymity, broad and persistent visibility, rapid dissemination, and repeated exposure, allowing its influence to extend beyond the original interaction (Tokunaga, 2010; Peebles, 2014). Review evidence in university populations suggests that cyberbullying and cyberstalking victimization are not uncommon and are typically associated with poorer emotional experiences, interpersonal difficulties, and reduced well-being, highlighting the stress-related nature of online victimization (Li et al., 2026; Agha and Kamran, 2024; Ugwu and Trejos-Castillo, 2025).

Within educational settings, the consequences of cyberbullying victimization may not remain limited to psychological discomfort, but may further spill over into academic functioning. Recent research has linked cyberbullying victimization not only to negative psychological outcomes such as depression, anxiety, and reduced self-worth, but also to academic burnout, lower learning efficiency, and poorer use of cognitive and metacognitive learning strategies (Lu et al., 2025; Solas-Mart́ınez et al., 2025; Zhou et al., 2025). In other words, the consequences of cyberbullying victimization are not restricted to emotional distress; they may also affect how students cognitively engage with, sustain effort toward, and cope with academic tasks. Linking cyberbullying victimization to educational outcomes is therefore both empirically grounded and theoretically plausible within a digital-stress framework.

Perceived academic stress refers to students' appraisal of academic expectations, workload, examinations, time pressure, and other study-related demands (Bedewy and Gabriel, 2015). Consistent with the transactional model of stress, stress is more likely when perceived demands are appraised as exceeding the individual's available coping resources (Lazarus and Folkman, 1984). Recent higher-education research has consistently shown that academic stress is one of the most salient stressors during college years and is further associated with poorer mental well-being and greater academic burnout (Zhang et al., 2025). Academic stress is therefore not a peripheral variable in educational settings, but a key link between psychological adjustment and academic adaptation.

This issue may be especially salient among vocational college students. Compared with students in more traditional academic environments, vocational students often need to cope simultaneously with coursework, skills training, practicum tasks, and internship-related requirements. Their learning context is therefore more practice-oriented and may involve a heavier combination of parallel tasks, performance demands, and time constraints. Recent studies on higher vocational students' academic adjustment and mental-health-related functioning suggest that this population faces distinctive contextual pressures and adaptation challenges (Zhu et al., 2025; Li et al., 2025). Under such conditions, once cyberbullying victimization is added as an extra social stressor, students may become even more likely to perceive academic demands as difficult to manage.

Based on this reasoning, the present study assumes that cyberbullying victimization may not only generate general negative psychological consequences, but may also heighten vocational college students' perceptions of academic strain. Put differently, when students experience more frequent online attacks, exclusion, humiliation, or harassment, they may become more likely to appraise current coursework, performance expectations, and time demands as stressful and overwhelming. Accordingly, H1 is proposed: Cyberbullying victimization positively predicts perceived academic stress.

### 2.2 Cyberbullying victimization, anxiety, and self-control

The association between cyberbullying victimization and anxiety has both strong theoretical plausibility and growing empirical support. Cybervictimization is often accompanied by exposure to negative evaluation, relational exclusion, persistent worry, and anticipation of repeated harm. These features make it more than a discrete digital event; rather, it constitutes a stress experience marked by heightened vigilance and social threat. Recent systematic review and meta-analytic structural equation modeling work has identified internalizing problems as one of the central domains linking cyberbullying victimization to maladaptive outcomes, with anxiety and depression repeatedly positioned as key mechanisms and consequences. Research in college populations has likewise continued to connect cybervictimization with anxiety, depression, learning difficulties, and poorer adjustment, suggesting that anxiety may be understood as one of the most representative emotional responses to cyberbullying victimization (Elshaer et al., 2026; Liu and Liu, 2025).

From the perspective of stress and cognitive appraisal, the tendency for anxiety to emerge after cyberbullying victimization is closely related to the characteristics of online contexts themselves. Online attacks are often repeatedly visible, rapidly disseminated, difficult to predict in audience size, and relatively anonymous (Peebles, 2014). These features can intensify ongoing worry, sensitivity to social evaluation, and threat anticipation. For college students, such prolonged psychological activation may not only shape emotional experience but also weaken attentional stability and the ability to allocate resources effectively within academic settings (Sergeeva and Zheltukhina, 2025). In this sense, cyberbullying victimization may be processed not simply as a past event, but as an unresolved threat, thereby heightening anxiety. On this basis, the first part of the present hypothesis is proposed: Cyberbullying victimization positively predicts anxiety.

Compared with anxiety, self-control is better understood as a self-regulatory resource. Recent studies among Chinese college students indicate that self-control is closely related to learning engagement, positive psychological resources, and academic functioning, whereas low self-control is more readily associated with academic procrastination, time-management difficulty, and weaker performance (Yang et al., 2024). At the same time, recent research on digital behavior has repeatedly positioned self-control as a central explanatory variable in the links between social media dependence, digital technostress, and academic maladjustment (Tang and He, 2025). From this perspective, self-control should not be viewed as an abstract personality label alone, but as a regulatory capacity that enables individuals to control impulses, resist temptations, and maintain behavior consistent with higher-order goals (Pan et al., 2023).

Linking cyberbullying victimization to self-control is primarily grounded in resource-depletion and self-regulation perspectives. Recent digital-stress research suggests that sustained technological and online demands can impair judgment and self-regulatory capacity in college students, whereas online self-control functions as an important protective resource (Hu et al., 2025). In parallel, college student studies centered on self-control indicate that anxiety and self-control are meaningfully connected, and that self-control is vulnerable under prolonged stress and emotional activation (Ye et al., 2025). Although direct evidence specifically showing that cyberbullying victimization reduces self-control remains more limited than the evidence for the anxiety pathway, it is theoretically reasonable to conceptualize cybervictimization as a digital stressor that may weaken behavioral regulation and resource management. Repeated experiences of online attack, humiliation, exclusion, and harassment may continuously consume cognitive and emotional resources, making it harder for students to sustain attention, inhibit impulses, and persist toward long-term academic goals. On this basis, the second part of the present hypothesis is proposed: Cyberbullying victimization negatively predicts self-control. Taken together, H2 is proposed: Cyberbullying victimization positively predicts anxiety and negatively predicts self-control.

### 2.3 Anxiety and insomnia

Anxiety and insomnia are closely and consistently linked, and this relationship is especially common in college populations. University life is typically characterized by multiple demands related to academics, social adjustment, and future planning, under which anxiety and sleep problems often co-occur. Systematic review and meta-analytic evidence based on undergraduate samples has shown moderate and stable associations between stress, poor sleep quality, and insomnia symptoms, indicating that sleep problems should not be understood as isolated physiological phenomena but rather as part of broader patterns of psychological strain and emotional dysregulation. In college students, insomnia is therefore both a common concern and an important risk indicator of maladjustment (Gardani et al., 2022).

Mechanistically, anxiety is likely to contribute to insomnia because it involves persistent worry, heightened vigilance, and difficulty relaxing (Mellman, 2006). Under anxious states, individuals are more likely to engage in repetitive threat-related thinking, maintain high physiological arousal, and experience marked cognitive and emotional activation before sleep. These features can directly interfere with sleep initiation and also weaken sleep maintenance and restorative functioning. Recent research in college populations further suggests that trait anxiety is significantly associated with overall sleep quality and multiple sleep dimensions, with higher anxiety levels corresponding to poorer sleep experiences, longer sleep latency, and more pronounced daytime dysfunction (Aschale Wale et al., 2024).

Within university settings, this relationship is reflected not only in general correlations but also in differential insomnia risk. Recent undergraduate research has identified anxiety symptoms as a significant correlate of insomnia (Juwita et al., 2025). Likewise, in a recent Chinese university student study, students with poorer sleep quality showed substantially higher rates of anxiety symptoms, and this pattern remained evident after controlling for relevant covariates (Shi et al., 2025). These findings suggest that anxiety and sleep problems do not merely coexist by chance; rather, they form a continuing psychological–physiological linkage.

It should be noted that the relationship between anxiety and sleep is sometimes discussed as bidirectional in the literature. In other words, anxiety may impair sleep, while sleep disturbance may also intensify anxiety. Recent college student research has likewise emphasized the close coupling between the two (Zhong et al., 2025). Nevertheless, in the present theoretical framework, anxiety is positioned prior to insomnia for two reasons. First, the present study focuses on how cyberbullying victimization may influence subsequent adaptation through emotional processes, and anxiety is conceptually better suited as the more immediate emotional response to online victimization. Second, recent work on victimization and sleep problems has indicated that emotional mechanisms are central channels linking victimization to impaired sleep, with anxiety functioning as a particularly relevant intermediate process (Wang et al., 2025; Liu et al., 2026). Within the current model, treating anxiety as an antecedent of insomnia is therefore both theoretically coherent and consistent with the logic of serial mediation.

Taken together, evidence from college mental health research, sleep research, and victimization-related mechanisms supports the role of anxiety as an antecedent of insomnia. Higher anxiety is likely to increase difficulties in falling asleep, maintaining sleep, and obtaining restorative rest, thereby contributing to more severe insomnia symptoms. Accordingly, H3 is proposed: Anxiety positively predicts insomnia.

### 2.4 Insomnia, self-control, and perceived academic stress

Insomnia reflects not only disrupted sleep during the night, but also reduced resources for daytime functioning, attentional maintenance, and stress tolerance. Among college students, insufficient sleep or poor sleep quality has been associated with difficulties in classroom concentration, task efficiency, emotional functioning, and coping with academic demands (Gardani et al., 2022; Hershner and Chervin, 2014). Recent higher-education research has consistently linked sleep problems to students' stress experience, academic functioning, and overall quality of life. The more severe the sleep-related impairment, the more likely students are to perceive heavier burdens in relation to coursework, time demands, and everyday functioning (Alotaibi et al., 2020). In this sense, insomnia should not be viewed as an isolated health problem alone; it may also amplify students' subjective academic stress by weakening their restorative resources.

From an educational perspective, perceived academic stress fundamentally depends on whether students appraise academic demands as exceeding their available coping resources. Once sleep is disrupted, students are likely to experience lower energy restoration, weaker attentional control, reduced cognitive flexibility, and poorer tolerance for demanding tasks. Under such conditions, coursework, assessments, and scheduling demands may be more readily interpreted as overwhelming burdens. Recent studies on college students' sleep and stress have shown that sleep quality is closely related to stress levels, daytime functioning, and academic outcomes (Ruiz-Camacho and Gozalo, 2025). In higher education settings, sleep problems are therefore intertwined not only with mental health but also with the way students function academically (Pérez-Jorge et al., 2025). This pattern may be even more salient among vocational college students, whose academic lives often involve parallel demands from coursework, skills training, and practicum-related tasks, making them especially vulnerable to the stress-enhancing effects of sleep disruption. On this basis, the present study assumes that insomnia will heighten perceived academic stress.

In contrast to insomnia, self-control represents a more self-regulatory and goal-management-oriented personal resource. Recent research on Chinese college students has continued to link self-control to learning engagement, positive emotional resources, and academic adaptation, whereas deficient self-control is more readily associated with academic procrastination, task delay, and reduced learning efficiency (Zhao et al., 2025; Soumeya et al., 2024). In digital behavior research, self-control has also been repeatedly positioned as a key explanatory variable in maladaptive academic outcomes. For example, in the association between social media dependence and academic procrastination, lack of self-control has been identified as an important mediating process (Álvarez-Maldonado et al., 2024).

Linking self-control to perceived academic stress is supported by a clear resource-regulation logic. Academic stress does not depend solely on the objective amount of work; it also depends on whether students can organize their behavior effectively, manage time, and remain engaged with academic goals (Wolters and Brady, 2021). When self-control is strong, students are more likely to delay immediate gratification, reduce distractive behaviors, and sustain long-term goal pursuit. As a result, even under high demands, they may be less likely to interpret those demands as unbearable pressure. By contrast, weak self-control undermines planning and execution, making students more prone to procrastination, attentional fragmentation, and disorganized coping, thereby intensifying subjective academic stress (Duckworth et al., 2019). This resource function may be particularly important for vocational college students, whose learning environments are structured around simultaneous coursework, skills training, and practice-oriented requirements. Accordingly, the present study further assumes that self-control will be negatively associated with perceived academic stress.

Taken together, both insomnia and self-control provide important explanatory pathways for perceived academic stress. Insomnia may function as a risk factor that weakens restorative capacity and intensifies stress appraisals, whereas self-control may function as a protective resource that buffers students against academic demands. Therefore, H4 is proposed: Insomnia positively predicts perceived academic stress, whereas self-control negatively predicts perceived academic stress.

### 2.5 The serial mediating roles of anxiety and insomnia

From a process perspective, the effect of cyberbullying victimization on academic stress is unlikely to occur in a single step. Rather, it is more plausibly transmitted through a sequence of interconnected psychological and functional mechanisms. Recent systematic review and meta-analytic structural equation modeling work on cyberbullying victimization has increasingly converged on the view that the association between victimization and maladjustment is rarely purely direct; instead, it is often transmitted through intermediate processes such as internalizing problems, emotional dysregulation, and life stressors (Li et al., 2026; Garaigordobil and Machimbarrena, 2019). This suggests that explaining how cyberbullying victimization spills over into perceived academic stress requires more than a simple victimization–outcome linkage and calls for attention to the emotional and functional pathways unfolding in sequence.

Within this framework, anxiety and insomnia form a theoretically coherent sequential mechanism. As argued in the previous sections, cyberbullying victimization is likely to trigger persistent worry, sensitivity to social evaluation, and heightened threat anticipation, making anxiety a salient emotional response to online victimization. Anxiety, in turn, may interfere with sleep initiation and sleep maintenance through cognitive arousal, difficulty relaxing, and sustained physiological vigilance. Accordingly, anxiety and insomnia should not be treated as two isolated outcomes, but rather as successive stages within a continuous process: cybervictimization first activates anxiety, and anxiety subsequently undermines sleep and contributes to more pronounced insomnia symptoms. Recent work on college students' victimization and sleep problems is consistent with this reasoning, as emotional processes are repeatedly positioned as central pathways linking victimization to sleep impairment, with anxiety emerging as one of the most common key mechanisms (Morgan et al., 2025).

When this sequential mechanism is further situated in an academic context, its logic becomes even clearer. Anxiety itself can undermine attentional stability and students' ability to cope with academic tasks, whereas insomnia further reduces restorative capacity, daytime functioning, and tolerance for stress. When these two processes occur in sequence, students may become more likely to appraise coursework, assessment demands, time constraints, and practicum requirements as difficult to manage. In this sense, anxiety may be understood as the initial emotional activation following cyberbullying victimization, whereas insomnia may be understood as the extension of that activation into physiological and functional impairment. When this sequence continues, the downstream outcome may be heightened perceived academic stress. Recent studies of college student samples indicate that sleep quality is closely intertwined with stress experience, functioning, and broader academic adjustment (Dagani et al., 2025; Chen et al., 2025), reinforcing the educational-psychological plausibility of modeling anxiety and insomnia as a serial pathway.

Taken together, the preceding arguments support a serial indirect pathway in which cyberbullying victimization is associated with greater anxiety, anxiety is associated with more severe insomnia, and insomnia is associated with higher perceived academic stress. This specification is also consistent with previous chain-mediation research suggesting that the associations between cybervictimization and adjustment outcomes may unfold through successively linked intermediate processes (Luo et al., 2023). Accordingly, H5 is proposed: Anxiety and insomnia jointly play a serial mediating role in the relationship between cyberbullying victimization and perceived academic stress.

### 2.6 The mediating role of self-control

Self-control is typically understood as the capacity to regulate impulses and align behavior with longer-term goals (Tangney et al., 2004). In college populations, this capacity is relevant not only to general behavioral regulation but also to learning engagement, time management, and academic adaptation. Recent studies have consistently linked self-control to learning engagement among Chinese college students, while low self-control has been associated with maladaptive academic outcomes such as academic procrastination (Yue et al., 2024). In this sense, self-control should not be treated as an abstract personality label, but rather as an important academic resource directly involved in organizing behavior and allocating effort in learning contexts.

From a resource-regulation perspective, cyberbullying victimization may be associated with lower self-control because repeated online attack, exclusion, humiliation, or harassment can place continuing demands on the emotional and attentional resources required for effective self-regulation. Although direct longitudinal evidence for the specific pathway from cyberbullying victimization to self-control remains limited, related research on digital stress suggests that sustained online demands may undermine judgment and regulatory functioning (Hu et al., 2025). The proposed ordering from cyberbullying victimization to self-control should therefore be understood as a theory-driven specification rather than as an empirically established temporal sequence.

Self-control is positioned before perceived academic stress because it contributes to how students organize tasks, manage time, inhibit distractions, and sustain effort toward longer-term academic goals. Students with lower self-control are more likely to experience difficulties in time management, task initiation, attentional persistence, and academic procrastination (Wolters and Brady, 2021; Tang and He, 2025; Zhao et al., 2025). These regulatory difficulties may make coursework, assessments, skills training, and practicum requirements more likely to be appraised as exceeding the student's available coping resources. Accordingly, H6 is proposed: Self-control mediates the relationship between cyberbullying victimization and perceived academic stress. Given the cross-sectional design, reverse or reciprocal associations cannot be excluded.

Taken together, the present study assumes that cyberbullying victimization may not only exert a direct positive effect on vocational college students' perceived academic stress, but may also operate indirectly through two psychological pathways. On the one hand, cyberbullying victimization may heighten students' anxiety, which may further contribute to insomnia and ultimately increase perceived academic stress. On the other hand, cyberbullying victimization may undermine students' self-control, and reduced self-control may in turn intensify their perceptions of academic stress. On this basis, the present study develops a theoretical model that incorporates a direct effect, a serial mediating pathway involving anxiety and insomnia, and a mediating pathway involving self-control, from which H1–H6 are proposed to systematically examine the mechanisms linking cyberbullying victimization to perceived academic stress among vocational college students.

## 3 Methodology

### 3.1 Participants and procedure

This study collected data through a questionnaire survey administered to vocational college students in China, with participants mainly recruited from vocational institutions in Chongqing and Zhejiang Province. A convenience sampling strategy was adopted. Data were collected through an anonymous online questionnaire administered during designated pre-class periods at the participating vocational colleges. Before access to the questionnaire was provided, members of the research team briefly introduced the purpose and procedures of the study and emphasized that participation was entirely voluntary, that students could decline to participate or discontinue without penalty, and that no compensation was provided. With logistical assistance from teachers or student counselors at the participating institutions, the online questionnaire link was then made available to the students. Those who chose to participate completed the questionnaire independently within a designated period.

No personally identifying information was collected. The first page of the online questionnaire described the academic purpose of the study, the voluntary and anonymous nature of participation, the confidentiality of participants' responses, and their right to discontinue before submission. Electronic informed consent was obtained before participants proceeded to the questionnaire.

Two attention-check items were embedded in the online questionnaire to identify potentially careless or random responding. One was an instructed-response item requiring participants to select the cross symbol ( × ) rather than the check mark (✓), and the other was a simple arithmetic item asking participants to provide the answer to 8+2. A response was excluded if either attention-check item was answered incorrectly or if one or more substantive questionnaire items were left unanswered.

A total of 400 online questionnaires were submitted. Twenty-two submissions met at least one of these exclusion criteria and were removed before the substantive analyses, resulting in a final analytic sample of 378 and a valid-response rate of 94.50%. All retained participants were currently enrolled vocational college students. The questionnaire did not collect information on formal psychiatric diagnoses, current psychiatric treatment, or psychotropic medication use. These factors were therefore not used as exclusion criteria or included as covariates in the analyses. The Generalized Anxiety Disorder Scale and the Insomnia Severity Index assessed self-reported symptom severity and were not used to establish clinical diagnoses.

Regarding gender, 198 participants (52.38%) were female and 180 (47.62%) were male. In terms of age distribution, 83 participants were 18 years old (21.96%), 105 were 19 years old (27.78%), 99 were 20 years old (26.19%), and 91 were 21 years old (24.07%). Regarding academic major, 52 participants were from medical and health-related programs (13.76%), 69 from modern service programs (18.25%), 66 from art and design programs (17.46%), 32 from mechanical manufacturing programs (8.47%), 64 from education-related programs (16.93%), 65 from business and trade programs (17.20%), and 30 from electronic information programs (7.94%).

To reduce evaluation apprehension and the potential influence of common method bias, the questionnaire presented the different constructs in separate sections, and the instructions emphasized that there were no right or wrong answers and that participants should respond according to their actual experiences. The final structural model included 41 observed indicators and estimated 88 free parameters, yielding 773 degrees of freedom (*df*). An additional power analysis based on the root mean square error of approximation (RMSEA) was conducted using the noncentral chi-square (χ^2^) distribution. With *N* = 378, *df* = 773, α = 0.05, RMSEA_0_ = 0.05, and RMSEA_1_ = 0.08, the estimated statistical power exceeded 0.99, indicating that the available sample provided adequate power to distinguish close overall model fit from poor overall model fit. This power estimate refers to the overall model-fit test rather than to each individual structural path.

### 3.2 Measures

Before the main data collection, the full Chinese-language questionnaire was subjected to qualitative expert review and questionnaire pilot testing. Three experts with backgrounds in vocational education and psychology reviewed the questionnaire, with particular attention to the revised and study-developed sections, for construct relevance, wording clarity, contextual appropriateness for vocational college students, item redundancy, and potential overlap among constructs. Because no standardized quantitative expert-rating form or content-validity index was used, this procedure was treated as a qualitative expert review rather than as a formal quantitative content-validity assessment. The full questionnaire was subsequently pilot-tested with 50 vocational college students in 2025. None of the pilot participants were included in the final analytic sample. The pilot test focused on item comprehensibility, response-option clarity, questionnaire flow, completion burden, and contextual relevance and was intended as a feasibility and comprehensibility check rather than as an independent psychometric validation study. Measure-specific adaptation and item-development details are provided below and in Supplementary material.

#### 3.2.1 Cyberbullying victimization

Cyberbullying victimization was assessed using an ECIPQ-informed, revised and integrated research version based primarily on the victimization dimension of the European Cyberbullying Intervention Project Questionnaire (ECIPQ; Del Rey et al., 2015). The measure retained the original 11-indicator format, two-month reference period, and 0–4 frequency response structure, but the original items were not reproduced verbatim or retained in a one-to-one correspondence. Core behavioral domains—including insulting or hurtful communication, threats, online exclusion, rumors and reputational harm, privacy violations, humiliating or manipulated visual material, and identity misuse—were retained and contextually reworded. Closely related content was integrated to reduce redundancy, while additional contextual specificity was introduced for the unauthorized sharing of private conversations, repeated harassing messages, and persistent targeting through anonymous or hidden accounts. These revisions were intended to reflect contemporary online interactions among vocational college students. These retained and extended content domains are consistent with established cybervictimization measures addressing written–verbal victimization, visual victimization, online exclusion, impersonation, platform-neutral harassment, and the unauthorized sharing of private conversations (Álvarez-García et al., 2017; Topcu and Erdur-Baker, 2018; Šincek, 2021; Shapka and Maghsoudi, 2017). A detailed mapping of the original ECIPQ content to the final items is provided in Supplementary Table S5.

Participants reported how often they had experienced each behavior during the previous two months using a 5-point scale ranging from 0 (never) to 4 (more than once a week), with higher scores indicating greater cyberbullying victimization. In the present study, the measure demonstrated good internal consistency (Cronbach's α = 0.912; McDonald's ω = 0.912).

#### 3.2.2 Anxiety

Anxiety was measured using the 7-item Generalized Anxiety Disorder Scale (GAD-7) (Spitzer et al., 2006). The GAD-7 is a brief and widely used instrument for assessing generalized anxiety symptoms in both research and practice, and its psychometric adequacy has also been supported in Chinese university student samples (Zhang et al., 2021). Representative items included “Feeling nervous, anxious, or on edge” and “Not being able to stop or control worrying.” Participants responded based on their experiences over the past two weeks using a 4-point scale ranging from 0 (not at all) to 3 (nearly every day), with higher scores indicating higher levels of anxiety. In the present study, the scale demonstrated good internal consistency (Cronbach's α = 0.902; McDonald's ω = 0.903).

#### 3.2.3 Insomnia

Insomnia was measured using the Insomnia Severity Index (ISI) (Bastien et al., 2001). The ISI is a classic self-report measure designed to assess the severity of insomnia and its daytime consequences, and the original scale consists of seven items. The Chinese version has also demonstrated sound psychometric properties (Yu, 2010). The instrument covers difficulties with sleep initiation, sleep maintenance, early awakening, satisfaction with sleep, daytime impairment, and distress related to sleep problems. Representative items included “Difficulty falling asleep” and “To what extent do your sleep problems interfere with your daytime functioning?” Participants responded based on their sleep condition during the past 2 weeks using a 5-point response format (0–4), with higher scores indicating more severe insomnia. In the present study, the scale demonstrated good internal consistency (Cronbach's α = 0.914; McDonald's ω = 0.914).

#### 3.2.4 Self-control

Self-control was assessed using an eight-item BSCS-based research version. The original 13-item Brief Self-Control Scale (BSCS) was developed by (Tangney et al. 2004) as a brief measure of dispositional self-control. Subsequent research has examined the dimensionality and psychometric properties of the BSCS in Chinese university students (Fung et al., 2020). To reduce the overall questionnaire length and respondent burden, eight items were selected and, where necessary, contextually reworded. The final set represented resistance to temptation, deliberation before action, attentional control, behavioral inhibition, resistance to immediate gratification, concentration, goal-directed persistence, and impulsive action. Four negatively worded items were retained to balance response direction.

Previous research has provided psychometric support for shortened BSCS configurations in Chinese student populations, including an eight-item model (Liang et al., 2022). However, the exact eight-item configuration used in the present study was treated as a BSCS-based research version rather than as a verbatim reproduction of a previously validated standard short form. The final item wording, scoring direction, represented content, and item-selection rationale are presented in Supplementary Table S6.

Participants responded according to their general behavioral tendencies using a 5-point scale ranging from 1 (not at all like me) to 5 (very much like me). Items SC2, SC5, SC6, and SC8 were reverse coded, with higher scores indicating stronger self-control. In the present sample, standardized factor loadings ranged from 0.621 to 0.826, composite reliability was 0.893, and average variance extracted was 0.514, providing preliminary evidence of construct reliability and convergent validity for this research version. The research version also demonstrated good internal consistency (Cronbach's α = 0.891; McDonald's ω = 0.893).

#### 3.2.5 Perceived academic stress

Perceived academic stress was assessed using an eight-item, task- and workload-oriented research measure developed for the present study. The Perception of Academic Stress Scale (PAS; Bedewy and Gabriel, 2015) served as the primary conceptual reference for identifying relevant domains, including workload, examinations and assessments, time constraints, performance demands, and external academic expectations. The PAS was used to inform the content domains rather than as a source for direct item-by-item adaptation.

An initial pool of 12 Chinese-language items was developed to cover conventional academic demands and requirements relevant to vocational education, including coursework, skills training, internship, and practicum tasks. Following the qualitative expert review and full-questionnaire pilot testing described above, five preliminary items emphasizing worry, social comparison, tension, self-doubt, or prolonged emotional reactions were removed to reduce conceptual overlap with anxiety and adjacent constructs. Seven task- and workload-oriented items were retained or refined, and one item concerning clustered deadlines across courses or training tasks was added, resulting in the final eight-item research measure. Further details are provided in Supplementary Tables S3, S4.

Participants rated the items according to their overall academic experience during the current semester using a 5-point scale ranging from 1 (strongly disagree) to 5 (strongly agree), with higher scores indicating greater perceived academic stress. In the present study, the research measure demonstrated good internal consistency (Cronbach's α = 0.914; McDonald's ω = 0.916).

### 3.3 Data analysis

Data analysis was conducted in four stages using SPSS 27.0.1 and AMOS 24. First, the raw data were screened and preprocessed. In accordance with the questionnaire design, the reverse-coded items in the self-control scale were recoded to ensure consistent scoring directions across items. Mean scores for each construct were computed for descriptive statistics and bivariate correlation analyses. Pearson correlations were retained as the primary bivariate estimates to summarize the linear associations among the construct-level composite scores. For confirmatory factor analysis (CFA) and structural equation modeling (SEM), item-level indicators were used to define the latent constructs in AMOS.

Prior to confirmatory factor analysis and structural equation modeling, univariate distributional properties were evaluated by examining the skewness and kurtosis of the 41 observed indicators. Multivariate normality and potential multivariate outliers were assessed in AMOS using the multivariate kurtosis statistic and Mahalanobis distance, respectively. To address the observed departure from multivariate normality, a Bollen–Stine bootstrap test with 5,000 resamples was conducted to evaluate the exact fit of the final structural model (Collier, 2020). Bias-corrected bootstrap confidence intervals based on 5,000 resamples were separately used to evaluate the structural paths and indirect effects, and an outlier exclusion sensitivity analysis was also conducted. Detailed diagnostic and sensitivity-analysis results are reported in Supplementary Table S7. Given the positive skewness of the cyberbullying victimization score and the departure from strict multivariate normality, Spearman's rank-order correlations among the five construct-level composite scores were additionally calculated as a sensitivity analysis.

Multicollinearity among the simultaneous predictors of perceived academic stress was assessed using tolerance and variance inflation factor (VIF) values based on the construct mean scores. Tolerance values above 0.10 and VIF values below 5 were considered indicative of no substantive multicollinearity (Kim, 2019).

Because all focal variables were assessed using self-report questionnaires at a single time point, Harman's single-factor test was conducted to assess potential common method bias. All 41 observed items were entered simultaneously into an unrotated principal component analysis, and the number of components with eigenvalues greater than 1 and the proportion of variance explained by the first component were examined. A first-component variance below 40% was interpreted as indicating that no single component accounted for the majority of the total variance (Podsakoff et al., 2024).

Second, the internal consistency of each scale was examined, and confirmatory factor analysis (CFA) was performed to evaluate the adequacy of the measurement model. Internal consistency was assessed using both Cronbach's α and McDonald's ω. McDonald's ω was included because it is suitable for congeneric measures in which item loadings may differ and does not require the assumption of tau-equivalence underlying Cronbach's α (Dunn et al., 2014). Omega coefficients were calculated for each scale using the final scored items, including the reverse-coded self-control items. Values of Cronbach's α and McDonald's ω of 0.70 or higher were interpreted as indicating acceptable internal consistency (Dunn et al., 2014; Nunnally and Bernstein, 1994).

The hypothesized five-factor measurement model, comprising cyberbullying victimization, anxiety, insomnia, self-control, and perceived academic stress, was first tested. Alternative measurement models were then specified and compared with the hypothesized model to evaluate discriminant validity.

Model fit was evaluated jointly using the chi-square statistic divided by degrees of freedom (χ^2^/*df*), the goodness-of-fit index (GFI), adjusted goodness-of-fit index (AGFI), incremental fit index (IFI), Tucker–Lewis index (TLI), comparative fit index (CFI), and root mean square error of approximation (RMSEA), together with its 90% confidence interval and the *p* value for the test of close fit (PCLOSE). Values of χ^2^/*df* < 3, GFI, AGFI, IFI, TLI, and CFI ≥0.90, and RMSEA ≤ 0.08 were interpreted as indicating acceptable model fit. CFI and TLI values near or above 0.95 and RMSEA values near or below 0.06 were interpreted as indicating particularly good fit (Hu and Bentler, 1999; Schermelleh-Engel et al., 2003). A PCLOSE value greater than 0.05 indicated that the hypothesis of close fit (RMSEA ≤ 0.05) was not rejected. These cutoff values were treated as interpretive guidelines rather than rigid decision rules, and model fit was evaluated from the overall pattern of indices rather than from any single cutoff. Construct reliability and convergent validity were further evaluated using standardized factor loadings, composite reliability (CR), and average variance extracted (AVE). CR values of 0.70 or higher and AVE values of 0.50 or higher were used as conventional benchmarks for adequate construct reliability and convergent validity, respectively (Fornell and Larcker, 1981). Because the mediation model was estimated using latent constructs, CR and AVE were reported as supplementary indicators of construct reliability and convergent validity within the measurement model before interpreting the structural paths and indirect effects; they were not used as tests of mediation.

Third, after support for the measurement model had been established, structural equation modeling (SEM) was used to test the proposed hypotheses. In the structural model, cyberbullying victimization was specified as the exogenous variable, anxiety, insomnia, and self-control were specified as mediators, and perceived academic stress was specified as the outcome variable. The model simultaneously included the direct path from cyberbullying victimization to perceived academic stress, the serial mediating pathway through anxiety and insomnia, and the mediating pathway through self-control. The hypotheses were evaluated on the basis of standardized path coefficients and their statistical significance.

Finally, the mediating effects were further examined using a bias-corrected bootstrap procedure with 5,000 resamples to estimate indirect effects and their 95% confidence intervals. An indirect effect was considered significant when the corresponding confidence interval did not include zero. This procedure was used to further test both the serial mediating effect of anxiety and insomnia and the mediating effect of self-control.

## 4 Research results

### 4.1 Descriptive statistics and correlations

Preliminary data screening indicated no severe univariate non-normality among the 41 observed indicators, with skewness values ranging from −0.547 to 1.828 and kurtosis values ranging from −0.938 to 3.538. The cyberbullying victimization score showed a positively skewed distribution (*skewness* = 1.280), reflecting a concentration of responses at relatively low victimization frequencies. This distributional pattern was consistent with its standard deviation being slightly larger than its mean. Strict multivariate normality was not fully supported; accordingly, 95% bias-corrected bootstrap confidence intervals based on 5,000 resamples were used to evaluate the structural paths and indirect effects.

Mahalanobis-distance screening identified four potential multivariate outliers. Inspection of these cases revealed no out-of-range responses or apparent data-entry errors. Re-estimation of the identical structural model after temporarily excluding these cases yielded substantively unchanged model fit, standardized path estimates, path directions, and statistical conclusions. The full sample was therefore retained for the primary analyses. Detailed distributional diagnostics and comparisons between the full-sample and sensitivity models are provided in Supplementary Table S7.

Table 1 presents the descriptive statistics and Pearson correlations among the study variables. The correlations were in the hypothesized directions: cyberbullying victimization was positively associated with anxiety, insomnia, and perceived academic stress and negatively associated with self-control, whereas self-control was negatively associated with anxiety, insomnia, and perceived academic stress. Overall, the observed bivariate associations were consistent with the directions specified in the hypothesized structural model.

**Table 1:** Means, standard deviations, and correlations among the study variables.

Variable	M	SD	1	2	3	4	5
1. Cyberbullying victimization	0.604	0.616	1				
2. Self-control	3.481	0.841	–0.319**	1			
3. Anxiety	0.879	0.660	0.447**	–0.399**	1		
4. Insomnia	1.265	0.865	0.286**	–0.255**	0.594**	1	
5. Perceived academic stress	3.501	0.898	0.364**	–0.432**	0.459**	0.497**	1

*N* = 378. **p < 0.01.

As a sensitivity analysis addressing the observed distributional departures, Spearman's rank-order correlations were also calculated. Each of the 10 pairwise associations had the same direction as the corresponding Pearson correlation and remained statistically significant at *p* < 0.001. The absolute differences between the corresponding Pearson and Spearman coefficients ranged from 0.000 to 0.031, indicating that the substantive pattern of the bivariate associations was robust to the choice of correlation coefficient. The complete Spearman correlation matrix is provided in Supplementary Table S8.

Multicollinearity diagnostics for the simultaneous predictors of perceived academic stress indicated no substantive concern. Tolerance values ranged from 0.854 to 0.888, and VIF values ranged from 1.125 to 1.171.

Harman's single-factor test was conducted using an unrotated principal component analysis of all 41 observed items. Five components with eigenvalues greater than 1 were extracted, and the first unrotated component accounted for 31.497% of the total variance, which was below the 40% threshold (Podsakoff et al., 2024). These results indicated that no single component accounted for the majority of the observed variance, although common method bias could not be completely ruled out.

### 4.2 Measurement model and discriminant validity

Before testing the structural relationships, confirmatory factor analysis (CFA) was conducted to examine the adequacy of the measurement model. The hypothesized five-factor model showed good fit to the data, with χ^2^ = 825.607, *df* = 769, χ^2^/*df* = 1.074, *p* = 0.077, GFI = 0.907, AGFI = 0.896, IFI = 0.993, TLI = 0.993, CFI = 0.993, RMSEA = 0.014, 90% confidence interval (CI) [0.000, 0.021], and PCLOSE = 1.000. The χ^2^/*df*, GFI, IFI, TLI, CFI, RMSEA, and PCLOSE values met the fit criteria specified in Section 3.3. Although the AGFI value of 0.896 was marginally below the conventional 0.90 benchmark, the overall pattern of fit indices supported good fit for the hypothesized five-factor measurement model.

To further assess discriminant validity, several alternative measurement models were specified and compared with the hypothesized five-factor model. Specifically, a four-factor model combining anxiety and insomnia into a single factor was tested, followed by another four-factor model combining anxiety and perceived academic stress into one factor. In addition, a three-factor model combining anxiety, insomnia, and perceived academic stress into a single factor was examined. The model comparison results are presented in Table 2. Compared with the five-factor model, both four-factor models and the three-factor model showed substantially poorer fit. The four-factor model combining anxiety and insomnia yielded χ^2^ = 1497.703, *df* = 773, χ^2^/*df* = 1.938, CFI = 0.915, TLI = 0.910, and RMSEA = 0.050. The four-factor model combining anxiety and perceived academic stress showed an even poorer fit, with χ^2^ = 1832.259, *df* = 773, χ^2^/*df* = 2.370, CFI = 0.876, TLI = 0.868, and RMSEA = 0.060. The three-factor model combining anxiety, insomnia, and perceived academic stress demonstrated the poorest fit, with χ^2^ = 2494.021, *df* = 776, χ^2^/*df* = 3.214, CFI = 0.798, TLI = 0.787, and RMSEA = 0.077. Taken together, these findings indicated that the hypothesized five-factor model fit the data substantially better than the alternative models, supporting the empirical distinction among cyberbullying victimization, anxiety, insomnia, self-control, and perceived academic stress and providing evidence of discriminant validity.

**Table 2:** Comparison of alternative measurement models.

Model	χ^2^	*df*	χ^2^/*df*	GFI	AGFI	IFI	TLI	CFI	RMSEA	*Δχ* ^2^	*Δdf*
5-factor	825.607	769	1.074	0.907	0.896	0.993	0.993	0.993	0.014	–	–
4-factor A	1497.703	773	1.938	0.774	0.748	0.915	0.910	0.915	0.050	672.096	4
4-factor B	1832.259	773	2.370	0.708	0.674	0.876	0.868	0.876	0.060	1006.652	4
3-factor	2494.021	776	3.214	0.633	0.593	0.799	0.787	0.798	0.077	1668.414	7

χ^2^, chi-square; *df*, degrees of freedom; GFI, goodness-of-fit index; AGFI, adjusted goodness-of-fit index; IFI, incremental fit index; TLI, Tucker–Lewis index; CFI, comparative fit index; RMSEA, root mean square error of approximation; Δχ^2^, change in chi-square; Δ*df*, change in degrees of freedom. The five-factor model included cyberbullying victimization, anxiety, insomnia, self-control, and perceived academic stress. Four-factor model A combined anxiety and insomnia; four-factor model B combined anxiety and perceived academic stress; the three-factor model combined anxiety, insomnia, and perceived academic stress.

In addition, the reliability and convergent validity of the constructs were examined, and the results are presented in Table 3. The standardized factor loadings ranged from 0.589 to 0.827 for cyberbullying victimization, from 0.621 to 0.826 for self-control, from 0.601 to 0.839 for anxiety, from 0.696 to 0.845 for insomnia, and from 0.586 to 0.840 for perceived academic stress. Across the five measures, Cronbach's α coefficients ranged from 0.891 to 0.914, and McDonald's ω coefficients ranged from 0.893 to 0.916. All α and ω coefficients exceeded the 0.70 criterion specified in Section 3.3, indicating good internal consistency across the five measures. Composite reliability (CR) values ranged from 0.893 to 0.916 and therefore also exceeded the conventional 0.70 benchmark.

**Table 3:** Reliability and convergent validity of the constructs.

Construct	Standardized loading range	Cronbach's α	McDonald's ω	CR	AVE
Cyberbullying victimization	0.589–0.827	0.912	0.912	0.912	0.489
Self-control	0.621–0.826	0.891	0.893	0.893	0.514
Anxiety	0.601–0.839	0.902	0.903	0.903	0.573
Insomnia	0.696–0.845	0.914	0.914	0.914	0.605
Perceived academic stress	0.586–0.840	0.914	0.916	0.916	0.578

CR, composite reliability; AVE, average variance extracted.

The average variance extracted (AVE) values were 0.489 for cyberbullying victimization, 0.514 for self-control, 0.573 for anxiety, 0.605 for insomnia, and 0.578 for perceived academic stress. Thus, the AVE values for self-control, anxiety, insomnia, and perceived academic stress exceeded the conventional 0.50 benchmark, whereas the AVE for cyberbullying victimization was marginally below this benchmark. Given the composite reliability of 0.912, the standardized loading range of 0.589 to 0.827, and the overall support for the five-factor measurement model, the convergent validity evidence for cyberbullying victimization was considered broadly acceptable; nevertheless, its marginal AVE value was explicitly acknowledged and interpreted with appropriate caution.

Taken together, the CFA results, comparisons with the alternative measurement models, and the reliability and validity indices supported the overall adequacy of the five-factor measurement model. The results provided satisfactory reliability and convergent-validity evidence for self-control, anxiety, insomnia, and perceived academic stress, while indicating a marginal limitation in the convergent validity of the cyberbullying victimization construct. The measurement model was therefore considered adequate for the subsequent structural analyses, although findings involving cyberbullying victimization should be interpreted with appropriate caution.

### 4.3 Structural model and hypothesis testing

**Figure 1:**
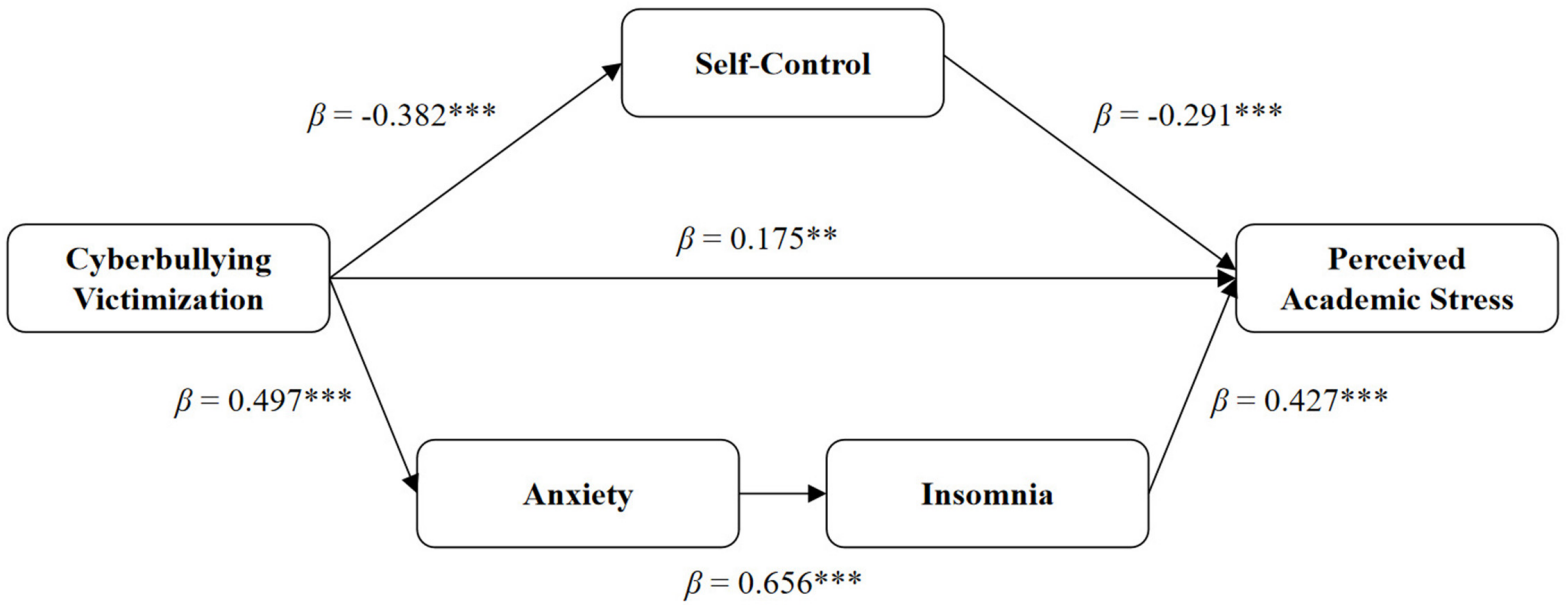
Structural equation model. ^**^p < 0.01; ^***^p < 0.001.

After support for the measurement model had been established, structural equation modeling was conducted to test the proposed hypotheses (Figure 1). The results indicated that the final structural model fit the data well, with χ^2^ = 858.031, *df* = 773, χ^2^/*df* = 1.110, GFI = 0.904, AGFI = 0.893, IFI = 0.990, TLI = 0.989, CFI = 0.990, RMSEA = 0.017, 90% CI [0.008, 0.023], and PCLOSE = 1.000. Although the AGFI value of 0.893 was marginally below the conventional 0.90 benchmark, the overall pattern of fit indices was favorable. Because model fit was evaluated jointly across multiple indices rather than on the basis of a single cutoff, the structural model was considered to demonstrate good overall fit, with the slightly subthreshold AGFI value acknowledged as a minor qualification. Given the observed departure from strict multivariate normality, the fit of the final structural model was additionally evaluated using a Bollen-Stine bootstrap test with 5,000 resamples. The test yielded a nonsignificant result (*p* = 0.432); therefore, the exact-fit null hypothesis was not rejected, providing additional support for the fit of the structural model under multivariate non-normality. All 5,000 bootstrap samples were successfully estimated. Although several fit indices were highly favorable, the structural model was strongly overidentified, with 773 degrees of freedom, rather than saturated or near-saturated. An audit of the final specification confirmed that the model contained no correlated residuals, no additional *post-hoc* structural paths, and no omitted hypothesized paths. The final model retained exactly the six theoretically specified structural paths. Moreover, the hypothesized five-factor measurement model showed substantially better fit than the alternative four-factor and three-factor models. Thus, the favorable fit was interpreted as support for the pre-specified model structure rather than as a consequence of adding data-driven parameters solely to improve model fit.

The standardized estimates, *p*-values, and bias-corrected bootstrap confidence intervals for all six hypothesized structural paths are presented in Table 4. Cyberbullying victimization was positively associated with anxiety and perceived academic stress and negatively associated with self-control. Anxiety was positively associated with insomnia, insomnia was positively associated with perceived academic stress, and self-control was negatively associated with perceived academic stress. All six structural paths were statistically significant, and none of the corresponding 95% bias-corrected bootstrap confidence intervals included zero.

**Table 4:** Structural path estimates.

Structural path	Standardized β	*p*	95% BC bootstrap CI
Cyberbullying victimization → Anxiety	0.497	< 0.001	[0.404, 0.575]
Anxiety → Insomnia	0.656	< 0.001	[0.570, 0.725]
Cyberbullying victimization → Self-control	−0.382	< 0.001	[−0.471, −0.285]
Insomnia → Perceived academic stress	0.427	< 0.001	[0.340, 0.515]
Self-control → Perceived academic stress	−0.291	< 0.001	[−0.387, −0.191]
Cyberbullying victimization → Perceived academic stress	0.175	0.001	[0.082, 0.266]

Standardized path coefficients are reported. BC bootstrap CI, bias-corrected bootstrap confidence interval. Confidence intervals were estimated using 5,000 bootstrap resamples.

With regard to hypothesis testing, H1 was supported because cyberbullying victimization was positively associated with perceived academic stress. H2 was supported because cyberbullying victimization was positively associated with anxiety and negatively associated with self-control. H3 was supported because anxiety was positively associated with insomnia. H4 was also supported because insomnia was positively associated with perceived academic stress, whereas self-control was negatively associated with perceived academic stress.

Regarding the mediating effects, the bias-corrected bootstrap results further showed that the total indirect effect of cyberbullying victimization on perceived academic stress was significant. Specifically, cyberbullying victimization was indirectly associated with perceived academic stress through the sequential pathway of anxiety and insomnia, and was also indirectly associated with perceived academic stress through self-control as a mediator. Taken together, the significance of the component paths and the bootstrap results suggest that anxiety and insomnia jointly played a serial mediating role between cyberbullying victimization and perceived academic stress, while self-control also functioned as a mediator. Therefore, both H5 and H6 were supported.

Importantly, after anxiety, insomnia, and self-control were included in the model, the direct association between cyberbullying victimization and perceived academic stress remained significant. This pattern was consistent with partial rather than full statistical mediation. In other words, the association between cyberbullying victimization and vocational college students' perceived academic stress was observed both directly and indirectly through the emotional–sleep and self-regulatory pathways, while additional unmeasured processes may also contribute to the remaining direct association.

Overall, the structural model results were largely consistent with the theoretical expectations, suggesting that the association between cyberbullying victimization and perceived academic stress among vocational college students is reflected in multiple pathways: a direct association, a serial indirect association through anxiety and insomnia, and an indirect association through self-control.

### 4.4 Mediation analysis

To further examine the statistical indirect associations, a bias-corrected bootstrap procedure with 5,000 resamples was conducted, and the results are presented in Table 5. The total effect of cyberbullying victimization on perceived academic stress was significant [estimate = 0.484, Boot SE = 0.059, 95% BC CI [0.377, 0.608]]. The direct effect was also significant [estimate = 0.199, Boot SE = 0.056, 95% BC CI [0.089, 0.307]], and the total indirect effect was significant as well [estimate = 0.285, Boot SE = 0.040, 95% BC CI [0.215, 0.372]]. These results indicate that cyberbullying victimization was associated with perceived academic stress both directly and indirectly.

**Table 5:** Direct, indirect, and total effects of cyberbullying victimization on perceived academic stress.

Path	Effect type	Estimate	Boot SE	95% BC bootstrap CI
CBV → PAS	Total effect	0.484	0.059	[0.377, 0.608]
CBV → PAS	Direct effect	0.199	0.056	[0.089, 0.307]
CBV → PAS	Total indirect effect	0.285	0.040	[0.215, 0.372]
CBV → ANX → INS → PAS	Specific indirect effect	0.158	0.028	[0.111, 0.223]
CBV → SC → PAS	Specific indirect effect	0.127	0.028	[0.079, 0.191]

CBV, cyberbullying victimization; PAS, perceived academic stress; ANX, anxiety; INS, insomnia; SC, self-control. Estimates are unstandardized effects. Boot SE, bootstrap standard error; BC bootstrap CI, bias-corrected bootstrap confidence interval. Specific indirect effects were estimated using a bias-corrected bootstrap procedure with 5,000 resamples and were calculated as products of the relevant unstandardized path coefficients. Table 4 reports standardized structural path coefficients, whereas the estimates in the present table are unstandardized effects; therefore, the numerical values in the two tables are not directly comparable.

Further decomposition showed that the serial indirect effect of cyberbullying victimization on perceived academic stress through anxiety and insomnia was significant [estimate = 0.158, Boot SE = 0.028, 95% BC CI [0.111, 0.223]]. The indirect effect through self-control was also significant [estimate = 0.127, Boot SE = 0.028, 95% BC CI [0.079, 0.191]]. Taken together, these findings indicate that both the serial pathway involving anxiety and insomnia and the pathway involving self-control contributed significantly to the mediating process.

Because both the direct effect and the total indirect effect remained significant, the findings support a partial mediation model.

## 5 Discussion

### 5.1 Major findings

The first major finding was that cyberbullying victimization was positively associated with perceived academic stress among vocational college students. This result extends university-level evidence showing that cyberbullying involvement is related to difficulties in study organization, examination preparation, adaptation to university, and broader academic adjustment (Aparisi et al., 2021; Peled, 2019). The present study adds to this literature by focusing specifically on students' subjective appraisal of academic burden rather than on academic performance or general university adaptation. From a demand–resource appraisal perspective, cyberbullying victimization may constitute an additional interpersonal burden that coincides with coursework, performance expectations, and time demands being experienced as more difficult to manage. Nevertheless, because the data were cross-sectional, the observed relationship should be interpreted as an association rather than as evidence that cyberbullying victimization causes academic stress.

The results also supported a serial indirect association involving anxiety and insomnia. Previous university research has shown that greater cybervictimization is associated with higher anxiety (Rahman et al., 2023). Separate research among university students has also demonstrated close associations between anxiety, insomnia, poor sleep quality, and daytime sleepiness (Choueiry et al., 2016). Considered together, these findings are consistent with the emotional–sleep pathway proposed in the present study. Cyberbullying victimization may coincide with persistent threat appraisal and heightened anxiety, while anxiety-related cognitive and physiological activation may be associated with greater difficulty initiating or maintaining sleep. Sleep-related impairment may then coincide with reduced restorative resources for managing academic demands. Thus, the observed serial association is consistent with a theoretically coherent account in which emotional and sleep-related difficulties may jointly link digital victimization with perceived academic stress. However, the proposed ordering of anxiety and insomnia remains theory-driven and cannot be established temporally or causally from the present cross-sectional data.

A separate indirect association was observed through self-control. Previous research among undergraduate students has reported a negative association between academic stress and self-regulation (Hj Ramli et al., 2018). Research with college students has also identified self-control as an important self-regulatory resource associated with lower perceived stress (Wayment and Cavolo, 2019). The present study extends this evidence by positioning self-control within the association between cyberbullying victimization and perceived academic stress. Students reporting weaker self-control may experience greater difficulty organizing tasks, resisting distractions, and sustaining effort toward longer-term academic goals, which may make academic demands appear less manageable. At the same time, the findings do not establish that cyberbullying victimization temporally reduces self-control or that lower self-control causes greater perceived academic stress. Reverse or reciprocal associations also remain possible.

The persistence of a significant direct association after anxiety, insomnia, and self-control were included indicates a partial rather than complete statistical mediation pattern. Previous university research has identified social support as another process involved in the psychological adjustment of students reporting cyberbullying victimization (Ho et al., 2020). This suggests that the correlates of cybervictimization are unlikely to be accounted for by a single pathway. Other factors not examined in the present model, including social support, coping style, school belonging, and negative cognitive appraisal, may therefore contribute to the remaining direct association. These additional processes should be examined using longitudinal or time-lagged designs.

Taken together, the findings should be interpreted as theoretically informed statistical associations rather than as evidence that the proposed pathways represent confirmed temporal or causal mechanisms. Within this interpretive boundary, the findings are consistent with complementary perspectives based on stress processes and self-regulation. The anxiety–insomnia pathway reflects the joint relevance of emotional activation and sleep-related functional difficulty, whereas the self-control pathway reflects variation in the regulatory resources associated with managing academic demands. The two pathways should therefore be understood as complementary rather than competing interpretations of the association between cyberbullying victimization and perceived academic stress.

### 5.2 Theoretical and practical implications

The significance of the present study lies in both its theoretical and practical implications.

#### 5.2.1 Theoretical implications

At the theoretical level, the present study extends the range of outcomes examined in cyberbullying victimization research. Much of the university-level evidence has examined adverse mental-health outcomes, including depression, anxiety, stress, and suicidal behavior (Arif et al., 2024). The present study moves this line of inquiry further into the educational context by examining perceived academic stress. This extension suggests that cyberbullying victimization may be associated not only with students' psychological adjustment but also with how they experience academic demands, performance expectations, and time pressure. By incorporating perceived academic stress into the explanatory framework, the present study strengthens the connection between cyberbullying research and educational psychology.

Second, the present study contributes to understanding the processes through which cyberbullying victimization may be associated with perceived academic stress by integrating two qualitatively different indirect pathways within a single model. The emotional–sleep pathway highlights the joint relevance of anxiety and insomnia, whereas the self-regulatory pathway highlights self-control as a regulatory resource associated with the management of academic demands. This distinction is compatible with conservation-of-resources theory, which proposes that stress is particularly likely when valued psychological or functional resources are threatened or lost (Hobfoll, 1989). In the present framework, anxiety and insomnia represent risk-related states associated with emotional and functional difficulty, whereas self-control represents a regulatory resource relevant to academic task management. Their simultaneous inclusion therefore suggests that the association between digital victimization and academic adjustment may involve both heightened risk-related states and variation in regulatory resources.

Third, the present study connects a stress-process perspective with an academic self-regulation perspective. The stress-process model emphasizes that stressors may be linked to adjustment outcomes through intervening strains and psychosocial resources (Pearlin et al., 1981), whereas academic self-regulation research emphasizes goal setting, strategic action, self-monitoring, and behavioral adjustment in learning contexts (Zimmerman, 2002). Applied to the present findings, the anxiety–insomnia pathway can be interpreted as a sequence of interrelated emotional and functional difficulties, while self-control can be interpreted as one regulatory resource associated with students' capacity to manage academic requirements. These perspectives converge on perceived academic stress while providing complementary rather than competing interpretations. The remaining direct association further indicates that these perspectives are not exhaustive. Future research may therefore incorporate additional variables, such as social support, school belonging, coping style, and negative cognitive appraisal, to develop a broader explanatory framework.

#### 5.2.2 Practical and clinical implications

At the practical and clinical levels, the present cross-sectional findings may offer preliminary implications for student support, campus counseling, and mental health services in vocational colleges. However, the findings do not demonstrate that any specific screening, prevention, counseling, or intervention strategy will produce beneficial outcomes.

At the service level, campus counselors or other qualified staff may consider asking students who seek help for persistent anxiety, sleep difficulties, or academic stress about recent experiences of cyberbullying victimization and their perceived effects on daily and academic functioning. Conversely, when cyberbullying victimization is disclosed, campus counselors or other qualified staff may consider assessing emotional distress, sleep-related difficulties, academic functioning, and problems with task management or behavioral regulation. These domains should inform a broader assessment and case formulation rather than be treated as evidence of a specific clinical diagnosis.

For students reporting persistent, severe, or functionally impairing symptoms, referral to appropriately qualified mental health or medical professionals may be considered. At the institutional level, vocational colleges may also consider linking cyberbullying reporting procedures with counseling, sleep-related guidance, and academic-skills or self-management support. Difficulties in self-control should not be interpreted as a psychiatric disorder or as a treatment target shown by the present study to reduce academic stress. Rather, they may indicate a need for support with time planning, task initiation, distraction management, and sustained effort. Where appropriate, coordination among campus counselors, academic advisors, student-affairs personnel, and online-safety or reporting channels may help address the psychological, academic, and digital-contextual aspects of students' concerns.

Because the present study did not evaluate diagnostic accuracy, clinical outcomes, or the effectiveness of any specific service model or intervention, these implications should be regarded as preliminary and hypothesis-generating. They identify potentially relevant domains for assessment, referral, and coordinated support rather than demonstrating that any particular strategy will improve student outcomes.

### 5.3 Limitations and future directions

Although the present study identified multi-path relationships among cyberbullying victimization, anxiety, insomnia, self-control, and perceived academic stress in a sample of vocational college students, several limitations should be acknowledged and addressed in future research.

First, this study employed a cross-sectional questionnaire design, and the findings therefore reflect statistical associations rather than strict causal relationships. Although the proposed model was grounded in a coherent stress-process and self-regulation framework, and the structural equation modeling results were consistent with the hypothesized directions, the relationships among cyberbullying victimization, anxiety, insomnia, self-control, and perceived academic stress may in reality be dynamic or even bidirectional. In particular, the present cross-sectional data cannot determine whether anxiety precedes insomnia, insomnia precedes anxiety, or reciprocal processes operate between the two constructs. Future research could adopt longitudinal designs, time-lagged designs, or cross-lagged panel models to test temporal ordering and causal mechanisms more rigorously.

Second, all data in the present study were based on students' self-reports, which may have introduced common method bias, social desirability bias, recall bias, and other forms of subjective reporting bias. Although anonymity, standardized response instructions, independent questionnaire completion, and two attention-check items were used to improve response quality, these procedures could not eliminate subjective reporting biases or all forms of careless responding.

Relatedly, the questionnaire did not assess formal psychiatric diagnoses, current psychiatric treatment, or psychotropic medication use. These unmeasured clinical and treatment-related factors may have influenced participants' self-reported anxiety, insomnia, self-control, and perceived academic stress and therefore represent potential sources of unmeasured confounding. Because the anxiety and insomnia instruments assessed symptom severity rather than providing clinical diagnoses, the presence of clinically significant, subclinical, or previously diagnosed conditions could not be determined. Future studies should incorporate multi-source or objective data where feasible, collect relevant clinical-history and treatment information, and examine whether the observed associations remain robust after accounting for these factors.

Third, the sample was drawn mainly from vocational colleges in Chongqing and Zhejiang, and the age range was concentrated between 18 and 21 years. The generalizability of the findings should therefore be interpreted with caution. Students from different regions, school types, or educational stages may differ in online behavior patterns, support environments, and the structure of academic stress. Future research could expand the sampling scope to include students from more diverse regions and institutions, and could also compare vocational college students with students from more traditional university settings in order to examine the stability and applicability of the present model across educational contexts.

Fourth, although demographic information such as gender, age, and academic major was collected and reported to describe the sample, these variables were not included as covariates in the primary structural model. Nor were formal subgroup or multi-group analyses conducted to examine whether the proposed pathways differed across demographic groups. Therefore, the present findings should be interpreted as overall associations observed in the full sample. Future research could further test the robustness of the model by incorporating theoretically relevant demographic covariates or by conducting multi-group analyses across different student subgroups.

Fifth, although the perceived academic stress measure underwent qualitative expert review and pilot testing and showed satisfactory measurement properties in the present sample, its psychometric properties require further evaluation in independent and more diverse vocational college samples.

Despite these limitations, the present study still provides meaningful evidence for understanding the association between cyberbullying victimization and perceived academic stress among vocational college students. Further extensions in research design, sampling, measurement, and mechanism modeling may help reveal the complex relationship between digital victimization experiences and educational adjustment more systematically.

## 6 Conclusion

This study examined the association between cyberbullying victimization and perceived academic stress among Chinese vocational college students and further tested the roles of anxiety, insomnia, and self-control within this framework. The findings showed that cyberbullying victimization was positively associated with perceived academic stress and was also indirectly associated with academic stress through a serial pathway involving anxiety and insomnia, as well as through self-control. In other words, the relationship between cyberbullying victimization and academic adjustment appeared to be reflected in both heightened risk-related states and weakened regulatory resources.

Overall, the present study suggests that cyberbullying victimization may be understood not only as an interpersonal problem in digital settings, but also as a risk-related experience linked to students' academic adjustment. By identifying its multi-path association with perceived academic stress among vocational college students, this study provides more fine-grained evidence for understanding the relationship between digital victimization and educational adjustment. At the same time, the findings should be interpreted with appropriate caution given the cross-sectional and self-report nature of the data. Future longitudinal and intervention-based studies are needed to examine causal relationships and to determine whether the pathways identified here can be translated into effective school-based support strategies.

## Data Availability

The raw data supporting the conclusions of this article will be made available by the authors, without undue reservation.

## References

[B1] Agha F. Kamran F. (2024). Cyber-stalking, social comparison, and psychological well-being in university students. Appl. Psychol. Rev. 3, 1–18. doi: 10.32350/apr.31.01

[B2] Alotaibi A. D. Alosaimi F. M. Alajlan A. A. Abdulrahman K. A. B. (2020). The relationship between sleep quality, stress, and academic performance among medical students. J. Fam. Community Med. 27, 23–28. doi: 10.4103/jfcm.JFCM_132_19 32030075 PMC6984036

[B3] Álvarez-García D. Núñez J. C. Barreiro-Collazo A. García T. (2017). Validation of the cybervictimization questionnaire (cyvic) for adolescents. *Comput. Human Behav*. 70, 270–281.

[B4] Álvarez-Maldonado D. Barrientos-Oradini N. Araneda-Reyes M. Aparicio-Puentes C. Cofré-Sandoval F. (2024). Self-control and academic performance. A comprehensive review and empirical study. Encuentros 22, 43–57.

[B5] Aparisi D. Delgado B. Bo R. M. Martínez-Monteagudo M. C. (2021). Relationship between cyberbullying, motivation and learning strategies, academic performance, and the ability to adapt to university. Int. J. Environ. Res. Public Health 18:10646. doi: 10.3390/ijerph182010646 34682391 PMC8535497

[B6] Arif A. Qadir M. A. Martins R. S. Khuwaja H. M. A. (2024). The impact of cyberbullying on mental health outcomes amongst university students: a systematic review. PLoS Mental Health 1:e0000166. doi: 10.1371/journal.pmen.0000166 41661848 PMC12798282

[B7] Aschale Wale M. Reta Y. Addis H. Tarekegn R. Tafese M. Tsega Chekol A. (2024). Predictors of insomnia among undergraduate students at Hawassa University Sidama, Ethiopia, 2023: a facility-based cross-sectional study. Front. Psychiatry 15:1352291. doi: 10.3389/fpsyt.2024.1352291 39220181 PMC11363190

[B8] Barbayannis G. Bandari M. Zheng X. Baquerizo H. Pecor K. W. Ming X. (2022). Academic stress and mental well-being in college students: correlations, affected groups, and COVID-19. Front. Psychol. 13:886344. doi: 10.3389/fpsyg.2022.886344 35677139 PMC9169886

[B9] Bastien C. H. Vallières A. Morin C. M. (2001). Validation of the insomnia severity index as an outcome measure for insomnia research. Sleep Med. 2, 297–307. doi: 10.1016/S1389-9457(00)00065-4 11438246

[B10] Bedewy D. Gabriel A. (2015). Examining perceptions of academic stress and its sources among university students: the perception of academic stress scale. Health Psychol. Open 2:2055102915596714. doi: 10.1177/2055102915596714 28070363 PMC5193280

[B11] Bussu A. Pulina M. Ashton S.-A. Mangiarulo M. Molloy E. (2025). Cyberbullying and cyberstalking victimisation among university students: a narrative systematic review. Int. Rev. Victimol. 31, 59–90. doi: 10.1177/02697580241257217

[B12] Chen C. Liu K. Zhou Z. (2025). How depression affects school social adaptation: the mediating role of sleep quality and the buffering effect of physical activity. Front. Sports Active Living 7:1716670. doi: 10.3389/fspor.2025.1716670 41357828 PMC12675383

[B13] Choueiry N. Salamoun T. Jabbour H. El Osta N. Hajj A. Rabbaa Khabbaz L. (2016). Insomnia and relationship with anxiety in university students: a cross-sectional designed study. PLoS ONE 11:e0149643. doi: 10.1371/journal.pone.0149643 26900686 PMC4762701

[B14] Collier J. E. (2020). Applied structural equation modeling using AMOS: Basic to advanced techniques. Routledge.

[B15] Dagani J. Buizza C. Ghilardi A. (2025). Exploring the role of sleep and physical activity in academic stress, motivation, self-efficacy, and dropout intention among italian university students. Eur. J. Investig. Health Psychol. Educ. 16:3. doi: 10.3390/ejihpe16010003 41590013 PMC12839884

[B16] Del Rey R. Casas J. A. Ortega-Ruiz R. Schultze-Krumbholz A. Scheithauer H. Smith P. . (2015). Structural validation and cross-cultural robustness of the european cyberbullying intervention project questionnaire. *Comput. Human Behav*. 50, 141–147.

[B17] Duckworth A. L. Taxer J. L. Eskreis-Winkler L. Galla B. M. Gross J. J. (2019). Self-control and academic achievement. Annu. Rev. Psychol. 70, 373–399. doi: 10.1146/annurev-psych-010418-103230 30609915

[B18] Dunn T. J. Baguley T. Brunsden V. (2014). From alpha to omega: a practical solution to the pervasive problem of internal consistency estimation. Br. J. Psychol. 105, 399–412. doi: 10.1111/bjop.12046 24844115

[B19] Elshaer I. A. Azazz A. M. Kooli C. Alyahya M. (2026). When support hurts: re-examining the cyberbullying victimization-mental health relationship among university students in Saudi Arabia. Eur. J. Investig. Health Psychol. Educ. 16:7. doi: 10.3390/ejihpe16010007 41590017 PMC12839641

[B20] Fang X. Wang F. Tian W. Liao J. Wei X. Lei L. (2026). How do traditional and cyber victimization affect sleep quality among college students? The chain mediating role of rumination and anxiety. J. Int. Violence 41, 3380–3399. doi: 10.1177/08862605251338780 40386823

[B21] Fornell C. Larcker D. F. (1981). Evaluating structural equation models with unobservable variables and measurement error. J. Market. Res. 18, 39–50. doi: 10.1177/002224378101800104

[B22] Fung S.-f. Kong C. Y. W. Huang Q. (2020). Evaluating the dimensionality and psychometric properties of the brief self-control scale amongst Chinese university students. Front. Psychol. 10:2903. doi: 10.3389/fpsyg.2019.02903 31969852 PMC6960225

[B23] Garaigordobil M. Machimbarrena J. M. (2019). Victimization and perpetration of bullying/cyberbullying: connections with emotional and behavioral problems and childhood stress. Psychosoc. Interv. 28, 67–73. doi: 10.5093/pi2019a3

[B24] Gardani M. Bradford D. R. Russell K. Allan S. Beattie L. Ellis J. G. . (2022). A systematic review and meta-analysis of poor sleep, insomnia symptoms and stress in undergraduate students. Sleep Med. Rev. 61:101565. doi: 10.1016/j.smrv.2021.101565 34922108

[B25] Hershner S. D. Chervin R. D. (2014). Causes and consequences of sleepiness among college students. Nat. Sci. Sleep 6, 73–84. doi: 10.2147/NSS.S62907 25018659 PMC4075951

[B26] Hj Ramli N. H. Alavi M. Mehrinezhad S. A. Ahmadi A. (2018). Academic stress and self-regulation among university students in malaysia: mediator role of mindfulness. Behav. Sci. 8:12. doi: 10.3390/bs8010012 29342910 PMC5791030

[B27] Ho T. T. Q. Li C. Gu C. (2020). Cyberbullying victimization and depressive symptoms in vietnamese university students: examining social support as a mediator. Int. J. Law Crime Justice 63:100422. doi: 10.1016/j.ijlcj.2020.100422

[B28] Hobfoll S. E. (1989). Conservation of resources: a new attempt at conceptualizing stress. Am. Psychol. 44:513. doi: 10.1037/0003-066X.44.3.513 2648906

[B29] Hu L.-t. and Bentler, P. M.. (1999). Cutoff criteria for fit indexes in covariance structure analysis: conventional criteria versus new alternatives. Struct. Equat. Model. Multidiscipl. J. 6, 1–55. doi: 10.1080/10705519909540118

[B30] Hu R. Gao Q. Hu R. Liu G. Zhang L. (2025). The relationship between digital technostress and cyber moral disengagement among college students: a moderated mediation model of psychological resilience, self-efficacy, and online self-control. Front. Psychol. 16:1706794. doi: 10.3389/fpsyg.2025.1706794 41307017 PMC12645413

[B31] Juwita C. P. Angela E. Napitupulu R. M. (2025). The association between anxiety disorders and insomnia among college students: a cross-sectional study. Contagion: Sci. Period. J. Public Health Coastal Health 7, 207–215. doi: 10.30829/contagion.v7i1.24131

[B32] Kim J. H. (2019). Multicollinearity and misleading statistical results. Korean J. Anesthesiol. 72, 558–569. doi: 10.4097/kja.19087 31304696 PMC6900425

[B33] Kwon M. Seo Y. S. Nickerson A. B. Dickerson S. S. Park E. Livingston J. A. (2020). Sleep quality as a mediator of the relationship between cyber victimization and depression. J. Nurs. Scholarsh. 52, 416–425. doi: 10.1111/jnu.12569 32510831 PMC7501234

[B34] Lazarus R. S. Folkman S. (1984). Stress, Appraisal, and Coping. Springer Publishing Company.

[B35] Li C. Lin S. Deng X. (2025). Mental health's shaping influence on college students' career choices: evidence from l university in Fujian Province, China. Front. Psychol. 16:1647718. doi: 10.3389/fpsyg.2025.1647718 41477614 PMC12752113

[B36] Li Y. Liu Y. Knoll M. Obsuth I. (2026). Mechanisms linking cyberbullying victimisation to internalising problems in youth: a systematic review and meta-analytic structural equation modelling. Clin. Psychol. Rev. 123:102672. doi: 10.1016/j.cpr.2025.102672 41330069

[B37] Liang W. Wang D. D. Shang B. R. Zhang C.-Q. Duan Y. P. Si G. Y. (2022). Further examination of the psychometric properties of the brief self-control scale: evidence from Chinese athletes and students. Int. J. Sport Exerc. Psychol. 20, 16–35. doi: 10.1080/1612197X.2020.1827000

[B38] Liu K. Wu D. Yang H. Zhang B. Li D. Yin H. (2026). The longitudinal relationship between bullying victimization and adolescents' sleep quality: a moderated mediation model. Psychol. Sch. 63, 727–738. doi: 10.1002/pits.70123

[B39] Liu X. Liu X. (2025). Cyberbullying victimization and suicidal ideation among college students: the mediating role of psychological pain and the moderating role of school bonding. BMC Psychiatry 25:564. doi: 10.1186/s12888-025-07007-8 40457227 PMC12131622

[B40] Lu Y. Dang L. Tao Y. (2025). The longitudinal influence of cyberbullying victimization on depression, self-esteem and academic burnout among chinese adolescents: mindfulness as a mediator. Front. Psychiatry 16:1607473. doi: 10.3389/fpsyt.2025.1607473 40405885 PMC12095141

[B41] Luo Q. Wu N. Huang L. (2023). Cybervictimization and cyberbullying among college students: the chain mediating effects of stress and rumination. Front. Psychol. 14:1067165. doi: 10.3389/fpsyg.2023.1067165 36844269 PMC9950563

[B42] Mellman T. A. (2006). Sleep and anxiety disorders. Psychiatr. Clin. 29, 1047–1058. doi: 10.1016/j.psc.2006.08.005 17118281

[B43] Morgan C. H. Stager L. M. Brockdorf A. N. Salamanca N. K. Amaya S. Mujica C. A. . (2025). Sleep-related concerns mediate the association between cyber-sexual victimization and psychological distress among diverse university students. Cyberpsychol. Behav. Soc. Netw. 28, 689–697. doi: 10.1177/21522715251375417 40928989 PMC12443332

[B44] Nunnally J. Bernstein I. H. (1994). Psychometric Theory, 3rd edn. McGraw-Hill.

[B45] Pan W. Long Y. Yue C. Tu S. Fang X. (2023). Identifying the individual differences of trait self-control: evidence from voxel-based morphometry. Pers. Individ. Dif. 202:111995. doi: 10.1016/j.paid.2022.111995

[B46] Pearlin L. I. Lieberman M. A. Menaghan E. G. Mullan J. T. (1981). The stress process. J. Health Soc. Behav. 22, 337–356. doi: 10.2307/2136676 7320473

[B47] Peebles E. (2014). Cyberbullying: hiding behind the screen. Paediatr. Child Health 19, 527–528. doi: 10.1093/pch/19.10.527 25587229 PMC4276384

[B48] Peled Y. (2019). Cyberbullying and its influence on academic, social, and emotional development of undergraduate students. Heliyon 5:e01393. doi: 10.1016/j.heliyon.2019.e01393 30963126 PMC6434491

[B49] Pérez-Jorge D. Boutaba-Alehyan M. González-Contreras A. I. Pérez-Pérez I. (2025). Examining the effects of academic stress on student well-being in higher education. Human. Soc. Sci. Commun. 12, 1–13. doi: 10.1057/s41599-025-04698-y

[B50] Podsakoff P. M. Podsakoff N. P. Williams L. J. Huang C. Yang J. (2024). Common method bias: It's bad, it's complex, it's widespread, and it's not easy to fix. *Annu. Rev. Organ. Psychol. Organ. Behav*. 11, 17–61. doi: 10.1146/annurev-orgpsych-110721-040030

[B51] Rahman T. Hossain M. M. Bristy N. N. Hoque M. Z. Hossain M. M. (2023). Influence of cyber-victimization and other factors on depression and anxiety among university students in bangladesh. J. Health Popul. Nutr. 42:119. doi: 10.1186/s41043-023-00469-0 37932869 PMC10629170

[B52] Ruiz-Camacho C. Gozalo M. (2025). Predicting university students' stress responses: the role of academic stressors and sociodemographic variables. Eur. J. Investig. Health Psychol. Educ. 15:163. doi: 10.3390/ejihpe15080163 40863285 PMC12385662

[B53] Schermelleh-Engel K. Moosbrugger H. Müller H. (2003). Evaluating the fit of structural equation models: tests of significance and descriptive goodness-of-fit measures. Methods Psychol. Res. 8, 23–74.

[B54] Sergeeva O. V. Zheltukhina M. R. (2025). Psychological well-being as a predictor of cyberbullying victimization in university students: a bayesian approach. Front. Educ. 10:1563122. doi: 10.3389/feduc.2025.1563122

[B55] Shapka J. D. Maghsoudi R. (2017). Examining the validity and reliability of the cyber-aggression and cyber-victimization scale. *Comput. Human Behav*. 69, 10–17.

[B56] Shi M. Miao R. Bing M. Liu S. (2025). The association between sleep quality and anxiety symptoms: a cross-sectional study based on tibetan university students at high altitude in China. Front. Psychol. 16:1505948. doi: 10.3389/fpsyg.2025.1505948 40226497 PMC11985792

[B57] Šincek D. (2021). The revised version of the committing and experiencing cyber-violence scale and its relation to psychosocial functioning and online behavioral problems. Societies 11:107.

[B58] Solas-Martínez J. L. la Torre-Cruz D. Manuel J. Rusillo-Magdaleno A. Martínez-López E. J. (2025). Bullying and cyberbullying is associated with low levels of cognitive and metacognitive learning strategies in young people. Front. Psychol. 16:1569400. doi: 10.3389/fpsyg.2025.1569400 40297593 PMC12034706

[B59] Soumeya M. Abdelhalim M. Mahassin I. (2024). Self-regulation, self-efficacy, and academic procrastination. Educ. Administr.: Theory Pract. 30, 942–952.

[B60] Spitzer R. L. Kroenke K. Williams J. B. Löwe B. (2006). A brief measure for assessing generalized anxiety disorder: the gad-7. Arch. Intern. Med. 166, 1092–1097. doi: 10.1001/archinte.166.10.1092 16717171

[B61] Staude-Müller F. Hansen B. Voss M. (2012). How stressful is online victimization? Effects of victim's personality and properties of the incident. Eur. J. Dev. Psychol. 9, 260–274. doi: 10.1080/17405629.2011.643170

[B62] Tang Y. He W. (2025). Impact of social media addiction on college students' academic procrastination: a chain mediated effect of lack of self-control and fear of missing out. Front. Psychol. 16:1668567. doi: 10.3389/fpsyg.2025.1668567 41190122 PMC12580371

[B63] Tangney J. P. Baumeister R. F. Boone A. L. (2004). High self-control predicts good adjustment, less pathology, better grades, and interpersonal success. J. Pers. 72, 271–324. doi: 10.1111/j.0022-3506.2004.00263.x 15016066

[B64] Tokunaga R. S. (2010). Following you home from school: a critical review and synthesis of research on cyberbullying victimization. Comput. Human Behav. 26, 277–287. doi: 10.1016/j.chb.2009.11.014

[B65] Topcu Ç. Erdur-Baker Ö. (2018). Rcbi-ii: The second revision of the revised cyber bullying inventory. Meas. Eval. Couns. Dev. 51, 32–41.

[B66] Ugwu K. O. Trejos-Castillo E. (2025). How cyberstalking victimization shapes academic and mental health outcomes in college students. Deviant Behav. 1–18. doi: 10.1080/01639625.2025.2537443

[B67] Wang J. Liu Y. Xiao T. Pan M. (2025). The relationship between bullying victimization and adolescent sleep quality: the mediating role of anxiety and the moderating role of difficulty identifying feelings. Psychiatry 88, 409–430. doi: 10.1080/00332747.2025.2484147 40192194

[B68] Wayment H. A. Cavolo K. (2019). Quiet ego, self-regulatory skills, and perceived stress in college students. J. Am. College Health 67, 92–96. doi: 10.1080/07448481.2018.1462826 29652612

[B69] Wolters C. A. Brady A. C. (2021). College students' time management: a self-regulated learning perspective. Educ. Psychol. Rev. 33, 1319–1351. doi: 10.1007/s10648-020-09519-z

[B70] Yang Y.-D. Zhou C.-L. Wang Z.-Q. (2024). The relationship between self-control and learning engagement among chinese college students: the chain mediating roles of resilience and positive emotions. Front. Psychol. 15:1331691. doi: 10.3389/fpsyg.2024.1331691 38445063 PMC10913274

[B71] Ye Z. Chi S. Ma X. Pan L. (2025). The impact of basic psychological needs on academic procrastination: the sequential mediating role of anxiety and self-control. Front. Psychol. 16:1576619. doi: 10.3389/fpsyg.2025.1576619 40463307 PMC12129888

[B72] Yu D. S. (2010). Insomnia severity index: psychometric properties with chinese community-dwelling older people. Journal of advanced nursing 66:2350–2359. doi: 10.1111/j.1365-2648.2010.05394.x 20722803

[B73] Yue L. Bakar Z. B. A. Ashari Z. (2024). Relationship between self-control and academic procrastination among college students in China: a general perspective. International Journal of Academic Research in Business and Social Sciences 14:419–427. doi: 10.6007/IJARBSS/v14-i3/21067

[B74] Zhang C. Wang T. Zeng P. Zhao M. Zhang G. Zhai S. . (2021). Reliability, validity, and measurement invariance of the general anxiety disorder scale among chinese medical university students. Front. Psychiatry 12:648755. doi: 10.3389/fpsyt.2021.648755 34093269 PMC8170102

[B75] Zhang J. Meng J. Wen X. (2025). The relationship between stress and academic burnout in college students: evidence from longitudinal data on indirect effects. Frontiers in Psychology 16:1517920. doi: 10.3389/fpsyg.2025.1517920 40491945 PMC12146318

[B76] Zhao X. Wang H. Ma Z. Zhang L. Chang T. (2025). Smartphone addiction and academic procrastination among college students: a serial mediation model of self-control and academic self-efficacy. Frontiers in psychiatry 16:1572963. doi: 10.3389/fpsyt.2025.1572963 40511461 PMC12158977

[B77] Zhong L. Ma X. Li S. Yu L. (2025). The relationship between trait anxiety and sleep quality in college students: an exploratory analysis of physical activity as a moderator. Front. Psychiatry 16:1563237. doi: 10.3389/fpsyt.2025.1563237 40547127 PMC12179125

[B78] Zhou W. Zhang Y. Xie B. Lu Y. (2025). Balancing the impact of cyberbullying on academic performance: the moderating role of sports engagement. Sage Open 15:21582440251401115. doi: 10.1177/21582440251401115

[B79] Zhu Y. Lu H. Wang X. Ma W. Xu M. (2025). The relationship between perceived peer support and academic adjustment among higher vocational college students: The chain mediating effects of academic hope and professional identity. Frontiers in Psychology 16:1534883. doi: 10.3389/fpsyg.2025.1534883 40062197 PMC11885234

[B80] Zimmerman B. J. (2002). Becoming a self-regulated learner: an overview. Theory into practice 41, 64–70. doi: 10.1207/s15430421tip4102_2

