# Supplementary material for: Cyberbullying victimization and perceived academic stress among vocational college students: the mediating roles of anxiety, insomnia, and self-control
Source: Front Psychol. 2026 Aug 26;17:1865591. doi: 10.3389/fpsyg.2026.1865591 (PMC13553904; doi:10.3389/fpsyg.2026.1865591)
Supplement: Supplementary file 1 [file Data_Sheet_1.PDF]

Supplementary Table S1. Construct boundaries and item-development rationale

Construct	Construct boundary	Main source / basis	Item-writing principle	Scale treatment in the present study
Cyberbullying victimization	Online victimization experiences, including hostility, exclusion, threat, repeated harassment, privacy violation, and identity misuse in digital contexts	ECIPQ victimization dimension	Wording adapted to fit contemporary college online interaction contexts, including social media, chat apps, and group chats	Revised and integrated research version; not a verbatim reproduction
Anxiety	Recent anxiety symptoms	GAD-7	Standard symptom-focused wording retained	Standard scale
Insomnia	Recent sleep difficulty severity, dissatisfaction, interference, noticeability, and distress	ISI	Standard wording retained	Standard scale
Self-control	Relatively stable self-regulatory capacity, including impulse regulation, attentional control, resistance to temptation, and long-term goal pursuit	BSCS	Selected a shorter set of items to reduce respondent burden while retaining core self-control facets	Abbreviated BSCS-based research version; includes reverse-coded items
Perceived academic stress	Task- and workload-oriented academic stress rather than general distress; covers workload, clustered deadlines, examinations/assessments, time burden, and performance demands	PAS and higher-education academic stress literature	Avoided overly affective wording to reduce overlap with anxiety; prioritized task demands and workload structure	8-item research version developed for the present study

Supplementary Table S2. Item provenance and treatment

Construct	Final item code	Final item (short label)	Source / basis	Treatment	Reason / rationale
Cyberbullying Victimization	CV1	insulting or hurtful comments online	ECIPQ victimization dimension	adapted wording	Updated for contemporary college online interaction contexts
Cyberbullying Victimization	CV2	rumors or false information online	ECIPQ victimization dimension	adapted wording	Retained the core meaning of online reputational victimization
Cyberbullying Victimization	CV3	threats or intimidation via online messages	ECIPQ victimization dimension	adapted wording	Preserved the threatening/ aggressive victimization facet
Cyberbullying Victimization	CV4	deliberate exclusion from group chats or online activities	ECIPQ victimization dimension	adapted wording	Updated to reflect student online social interaction settings
Cyberbullying Victimization	CV5	disclosure of private information without permission	ECIPQ victimization dimension	adapted and integrated wording	Captured privacy violation in a college-relevant online context
Cyberbullying Victimization	CV6	screenshotting/forwarding private conversations	ECIPQ victimization dimension	adapted and integrated wording	Made the item more specific to current digital communication practices
Cyberbullying Victimization	CV7	uploading humiliating photos or videos	ECIPQ victimization dimension	adapted wording	Retained humiliation-based online victimization content

Cyberbullying Victimization	CV8	editing/manipulating photos or videos to mock the target	ECIPQ victimization dimension	adapted and integrated wording	Captured digitally manipulated ridicule, which is common in current student contexts
Cyberbullying Victimization	CV9	impersonation or pretending to be the target online	ECIPQ victimization dimension	adapted wording	Preserved identity-misuse facet in online victimization
Cyberbullying Victimization	CV10	repeated harassing or disturbing messages	ECIPQ victimization dimension	adapted wording	Retained repeated online aggression facet
Cyberbullying Victimization	CV11	repeated targeting through anonymous or hidden accounts	ECIPQ victimization dimension	adapted and integrated wording	Added contextual specificity to current anonymous online harassment patterns
Self-control	SC1	resist temptation	BSCS	retained	Preserved resistance-to-temptation facet
Self-control	SC2	act before thinking things through	BSCS	retained; reverse-coded	Preserved impulse-control facet while balancing response direction
Self-control	SC3	stay focused on what is important	BSCS	retained	Represented attentional control facet
Self-control	SC4	stop oneself from inappropriate behavior	BSCS	retained	Preserved inhibitory control facet
Self-control	SC5	fun or pleasure interferes with	BSCS	retained; reverse-cod	Captured interference

		getting work done		ed	from immediate gratification
Self-control	SC6	difficulty concentrating on what should be done	BSCS	retained; reverse-coded	Represented distractibility and self-regulatory difficulty
Self-control	SC7	work steadily toward long-term goals	BSCS	retained	Preserved goal-directed persistence facet
Self-control	SC8	speak or act impulsively	BSCS	retained; reverse-coded	Preserved impulsive action facet
Perceived Academic Stress	AS1	academic tasks exceed manageable range	developed for the present study with reference to PAS and academic stress literature	newly written	Captured perceived workload burden rather than general distress
Perceived Academic Stress	AS2	examinations and assessments create substantial academic pressure	developed for the present study with reference to PAS and academic stress literature	newly written	Targeted examination - and assessment-related pressure
Perceived Academic Stress	AS3	insufficient time for assignments, coursework, or practical training	developed for the present study with reference to PAS and academic stress literature	newly written	Captured academic time burden
Perceived Academic Stress	AS4	pressure from grades, ranking, or performance demands	developed for the present study with reference to PAS and academic stress literature	newly written / integrated	Targeted performance-demand pressure in academic settings
Perceived Academic Stress	AS5	high expectations from teachers, parents, or others	developed for the present study with reference to PAS and academic stress literature	newly written	Represented external academic demand pressure
Perceived	AS6	academic tasks	developed for the	newly	Reflected

Academic Stress		take up time for rest and relaxation	present study with reference to PAS and academic stress literature	written	workload-related loss of rest time
Perceived Academic Stress	AS7	pressure from balancing coursework, skills training, and internship/practicum tasks	developed for the present study with reference to PAS and academic stress literature	newly written	Adapted academic stress content to the vocational college context
Perceived Academic Stress	AS8	clustered deadlines across courses or training tasks	developed for the present study with reference to PAS and academic stress literature	newly written	Specifically captured clustered deadlines and workload accumulation

Supplementary Table S3. Development and preliminary evaluation process of the perceived academic stress measure

Stage	Description
Conceptual specification	Perceived academic stress was defined as students' subjective appraisal of task demands and workload-related academic pressure rather than as general psychological distress. The intended content domains included workload, examinations and assessments, time constraints, performance requirements, external academic expectations, loss of rest time, and the accumulation of concurrent academic demands.

Primary conceptual basis	The Perception of Academic Stress Scale (PAS; Bedewy and Gabriel, 2015) served as the primary conceptual reference during the initial development process, particularly for the domains of workload, examinations, time constraints, pressure to perform, and external expectations. Additional academic-stress literature is cited in Supplementary Table S4 to document conceptual correspondence between the final item content and established academic-stressor domains; these citations do not imply direct item-by-item adaptation.
Initial item generation	A preliminary pool of 12 Chinese-language items was developed for vocational college students. The initial pool covered conventional coursework demands as well as skills training, internship, and practicum requirements relevant to vocational education.
Qualitative expert review	The preliminary Chinese-language items were qualitatively reviewed by three experts with backgrounds in vocational education and psychology. The review addressed construct relevance, wording clarity, contextual appropriateness, item redundancy, coverage of the intended academic-demand domains, and potential overlap with anxiety and adjacent constructs. The experts provided qualitative comments and revision suggestions. No standardized quantitative relevance-rating form or content-validity index was used; therefore, this procedure is described as a qualitative expert review rather than a formal quantitative content-validity assessment.
Questionnaire pilot testing	Before the main data collection, the full questionnaire was pilot-tested with 50 vocational college students in 2025. None of the pilot participants were included in the final analytic sample. The pilot test focused on item comprehensibility, response-option clarity, questionnaire flow, completion burden, and contextual relevance. It was intended as a questionnaire feasibility and comprehensibility check rather than as an independent factor-analytic or psychometric validation study.

Item revision	Following the expert consultation and pilot testing, five preliminary items emphasizing worry, social comparison, tension, self-doubt, or prolonged emotional reactions were removed to reduce conceptual overlap with anxiety or adjacent constructs. Seven task- and workload-oriented items were retained or refined, and one item concerning clustered deadlines across courses or training tasks was added.
Final measure	The revision process resulted in an eight-item, task- and workload-oriented research measure rated on a 5-point agreement scale, with higher scores indicating greater perceived academic stress.
Main-sample evaluation	In the final analytic sample, standardized factor loadings ranged from 0.586 to 0.840, Cronbach's alpha was 0.914, composite reliability was 0.916, and average variance extracted was 0.578. The hypothesized five-factor measurement model also fit substantially better than the alternative model combining anxiety and perceived academic stress, providing preliminary evidence of internal consistency, convergent validity, and empirical distinctiveness in the present sample.

Note. PAS = Perception of Academic Stress Scale. Initial item development, expert consultation, and questionnaire pilot testing were conducted in Chinese. English descriptions are provided for reporting purposes. References added to demonstrate conceptual correspondence should not be interpreted as evidence that the final items were directly translated or adapted from those measures.

Supplementary Table S4. Item-level revision pathway from the preliminary 12-item pool to the final eight-item perceived academic stress measure

Preliminary item	Revision decision	Final item	Revision rationale	Conceptual correspondence and supporting literature
Preliminary AS1. "I feel that my current academic tasks are more than I can comfortably handle."	Retained	Final AS1. "I feel that my current academic tasks are more than I can comfortably handle."	The item directly represented perceived workload relative to the student's perceived coping capacity and was consistent with the task- and workload-oriented construct definition.	Conceptually consistent with workload and excessive academic-demand content in the PAS and other academic-stressor measures (Bedewy and Gabriel, 2015; Kohn and Frazer, 1986; Sun et al., 2011).
Preliminary AS2. "I often worry that I may not meet course or academic requirements."	Removed	—	The item was framed primarily in terms of worry. Because worry was also directly represented in the anxiety measure, retaining this item could have increased conceptual overlap between perceived academic stress and anxiety.	Grade- and performance-related worry is represented in broader educational-stress research, including the ESSA, but the item was excluded here to maintain a narrower task- and demand-oriented construct boundary (Sun et al., 2011).

Preliminary AS3. "Exams, tests, or assessments often make me feel stressed."	Retained and refined	Final AS2. "Exams, tests, or assessments often place considerable academic pressure on me."	The wording was revised from a general emotional reaction ("make me feel stressed") to a more demand-focused appraisal of academic pressure.	Examination and assessment demands are established components of academic stress in the PAS and the Academic Stress Scale (Bedewy and Gabriel, 2015; Kohn and Frazer, 1986).
Preliminary AS4. "I feel that I do not have enough time to complete assignments, coursework, or practical training tasks."	Retained	Final AS3. "I feel that I do not have enough time to complete assignments, coursework, or practical training tasks."	The item directly represented academic time pressure and incorporated practical-training demands relevant to vocational college students.	Time constraints are represented in the PAS; the reference to practical training was a contextual extension for vocational education rather than a directly adapted PAS item (Bedewy and Gabriel, 2015).
Preliminary AS5. "I often feel pressure related to grades, ranking, or academic performance."	Retained and refined	Final AS4. "I often feel pressure related to grades, ranking, or performance requirements."	"Academic performance" was revised to "performance requirements" to emphasize perceived academic demands and to increase applicability across written, practical, and skills-based assessments.	Performance pressure and grade-related demands are represented in established academic-stress research (Bedewy and Gabriel, 2015; Sun et al., 2011).

Preliminary AS6. "I feel that teachers, parents, or others have high expectations for my academic performance."	Retained	Final AS5. "I feel that teachers, parents, or others have high expectations for my academic performance."	The item represented externally perceived academic expectations and was retained because it reflected a recognized source of academic pressure without directly measuring a general anxiety symptom.	Teacher and parental expectations are represented in the PAS and related educational-stress research (Bedewy and Gabriel, 2015).
Preliminary AS7. "I worry that my academic performance is worse than that of my peers."	Removed	—	The item combined social comparison with worry. It was less directly focused on the amount or structure of academic demands and could have introduced content related to comparison orientation and anxiety.	Social comparison may be relevant to academic stress, but it was not retained within the narrower task- and workload-oriented definition used in the present study.
Preliminary AS8. "I tend to feel tense when I think about academic tasks."	Removed	—	"Feeling tense" closely resembled affective and physiological anxiety wording. The item was removed to improve the conceptual distinction between perceived academic stress and anxiety symptoms.	The content was excluded on construct-boundary grounds rather than because academic tasks cannot elicit tension.

Preliminary AS9. “I sometimes doubt whether I can successfully complete my studies.”	Removed	—	The item primarily reflected academic self-doubt or perceived academic self-efficacy rather than the perceived magnitude or organization of academic demands.	Self-expectation and despondency appear in broader educational-stress measures, but these domains were outside the narrower task- and workload-oriented construct definition adopted here (Sun et al., 2011).
Preliminary AS10. “Academic tasks take up a great deal of my time for rest and relaxation.”	Retained	Final AS6. “Academic tasks take up a great deal of my time for rest and relaxation.”	The item captured the extent to which academic workload reduced time available for rest and recovery.	The PAS includes conceptually related content concerning whether sufficient relaxation time remains after academic work (Bedewy and Gabriel, 2015).
Preliminary AS11. “Balancing theory courses, skills training, internships, or practicum tasks is stressful for me.”	Retained	Final AS7. “Balancing theory courses, skills training, internships, or practicum tasks is stressful for me.”	The item was retained as a vocationally specific indicator of concurrent academic and practice-based demands. It extended conventional coursework-focused academic-stress content to the structure of vocational education.	This was a contextually developed item. No claim of direct adaptation from the PAS or another established scale is made.

Preliminary AS12. “Even small academic setbacks can make me feel stressed for quite a while.”	Removed	—	The item primarily reflected the duration of an emotional response to setbacks rather than the magnitude, timing, or structure of academic demands. It could also have captured general emotional reactivity.	The item was removed to preserve the distinction between academic demands and prolonged emotional responses.
No direct preliminary counterpart	Added during revision	Final AS8. “Deadlines from different courses or training tasks often cluster together, making my academic workload feel heavy.”	The item was added to represent the accumulation of simultaneous demands and clustered deadlines across coursework and training tasks. This content was considered particularly relevant in programs requiring parallel academic and practical assignments.	Overlapping assignment deadlines and workload management are represented within the academic-workload component of the Post-Secondary Student Stressors Index literature (Kuburi et al., 2025).

Note. Five preliminary items were removed, seven were retained or refined, and one item was added, resulting in the final eight-item measure. The revision rationales summarize the research team’s decisions following the qualitative expert consultation and questionnaire pilot testing. They should not be interpreted as verbatim statements or unanimous item-level judgments made by all three experts. The cited literature demonstrates conceptual correspondence with established academic-stress domains; it does not imply that the final items were directly translated or adapted item by item from these instruments.

Supplementary Table S5. Conceptual mapping of ECIPQ-related victimization content and contextual extensions to the final 11-item research version

ECIPQ-related content domain or contextual extension	Treatment in the present study	Final item(s)	Adaptation rationale and conceptual correspondence
Direct insulting or hurtful online communication	Retained and contextually reworded	CV1. Someone said insulting, mean, or hurtful things to me online.	Preserves the written–verbal hostility domain while using platform-neutral wording applicable to social media, chat applications, gaming platforms, and mobile messaging. Comparable written–verbal victimization content is represented in established cybervictimization measures (Del Rey et al., 2015; Álvarez-García et al., 2017; Shapka and Maghsoudi, 2017).
Harmful statements, rumors, or false information about the victim	Retained and integrated	CV2. Someone spread rumors or false information about me online.	Combines closely related forms of reputational victimization into one clearly interpretable indicator. Rumor spreading and written–verbal reputational harm are represented in established cybervictimization measures (Del Rey et al., 2015; Álvarez-García et al., 2017).

Threatening or intimidating online messages	Retained and contextually reworded	CV3. Someone threatened, intimidated, or tried to scare me through online messages.	Preserves the threat and intimidation domain while clarifying the behavioral form of the experience across contemporary communication channels (Del Rey et al., 2015; Álvarez-García et al., 2017).
Deliberate exclusion from online interaction	Retained and contextually reworded	CV4. Someone deliberately excluded me from group chats, online activities, or social interactions.	Updates online exclusion to reflect current student interaction through group chats, online activities, and platform-based social spaces. Online exclusion is an established cybervictimization domain (Del Rey et al., 2015; Álvarez-García et al., 2017).
Unauthorized access to, disclosure of, or use of private information	Retained, broadened, and integrated	CV5. Someone shared, forwarded, or disclosed my private information without permission.	Broadens the focus from a particular technical method, such as unauthorized account access, to the unauthorized disclosure, forwarding, or leaking of private information through contemporary digital channels. Related privacy-violation and information-sharing content appears in established and revised cybervictimization measures (Del Rey et al., 2015; Šincek, 2021).

Unauthorized sharing of private conversations or screenshots	Added as a contemporary contextual extension	CV6. Someone screenshotted, forwarded, or spread my private conversations without permission.	Captures the unauthorized taking, forwarding, or dissemination of screenshots and private conversations. Closely corresponding content concerning the unauthorized sharing of private-conversation screenshots appears in a revised cyber-violence measure (Šincek, 2021).
Posting embarrassing or humiliating photographs or videos	Retained and contextually reworded	CV7. Someone uploaded photos or videos that made me feel embarrassed or humiliated.	Preserves visual humiliation and the unauthorized distribution of embarrassing visual material. Similar visual-victimization content is represented in established cybervictimization measures (Del Rey et al., 2015; Álvarez-García et al., 2017; Shapka and Maghsoudi, 2017).
Altering photographs or videos to ridicule the victim	Retained and contextually specified	CV8. Someone edited, manipulated, or mocked my photos or videos to make fun of me.	Preserves manipulated-image victimization while clarifying contemporary image-editing and digitally altered ridicule. Related visual-victimization content is represented in established instruments (Del Rey et al., 2015; Álvarez-García et al., 2017).

Account-based impersonation, false identity creation, or pretending to be the victim	Integrated into broader identity-misuse content	CV9. Someone impersonated me or pretended to be me online.	Combines closely related forms of impersonation into one indicator to reduce redundancy and avoids restricting the behavior to account hacking alone. Impersonation is an established cybervictimization domain (Del Rey et al., 2015; Álvarez-García et al., 2017).
Repeated harmful, aggressive, or harassing electronic communication	Contextually extended and made explicit	CV10. Someone repeatedly sent me harassing, aggressive, or disturbing messages.	Makes repeated message-based harassment explicit and provides platform-neutral behavioral coverage. The use of technology-neutral wording is consistent with the development and revision rationale of updated cybervictimization instruments (Shapka and Maghsoudi, 2017; Topcu and Erdur-Baker, 2018).
Anonymity-enabled or hidden-identity harassment	Added as a contemporary contextual extension	CV11. Someone repeatedly targeted me through anonymous or hidden accounts.	Captures persistent targeting through anonymous or hidden accounts, reflecting the role of concealed identity in contemporary online harassment. This item is presented as a contextual extension rather than as a direct ECIPQ item or a direct adaptation from another established measure.

Note. ECIPQ = European Cyberbullying Intervention Project Questionnaire; CYVIC = Cybervictimization Questionnaire; CAV = Cyber-Aggression and Cyber-Victimization Scale; RCBI-II = second revision of the Revised Cyber Bullying Inventory. The original ECIPQ victimization dimension contains 11 items. The present measure retained an 11-indicator format, the two-month reference period, and the 0–4 frequency response structure, but it did not reproduce the original victimization items verbatim or maintain a one-to-one item correspondence. Some original content was retained and contextually reworded, closely related content was integrated, and several contemporary contextual indicators were added. The cited instruments demonstrate conceptual correspondence with established cybervictimization domains and do not imply that the final items were directly translated or adapted item by item from those instruments.

Supplementary Table S6. Item selection and treatment in the eight-item BSCS-based research version

Final item code	Final item wording	Scoring	Core self-control content	Selection rationale
SC1	I am good at resisting temptation.	Direct	Resistance to temptation	Selected to represent the ability to resist immediately attractive but potentially conflicting impulses.
SC2	I often act before thinking things through.	Reverse-coded	Deliberation and non-impulsivity	Selected to represent insufficient deliberation before action and the impulsive-action component of self-control.
SC3	I am able to stay focused on what is important.	Direct	Attentional control	Selected to represent the capacity to maintain attention on goal-relevant priorities.
SC4	I can stop myself from doing something inappropriate.	Direct	Behavioral inhibition	Selected to represent the capacity to inhibit behavior that is recognized as inappropriate.

SC5	Fun and enjoyment sometimes keep me from getting my work done.	Reverse-coded	Resistance to immediate gratification	Selected to represent interference from short-term enjoyment when completing necessary tasks.
SC6	I sometimes have difficulty concentrating on what I should do.	Reverse-coded	Concentration and distractibility	Selected to represent attentional difficulty and susceptibility to distraction during goal-directed behavior.
SC7	I am able to work steadily toward long-term goals.	Direct	Goal-directed persistence	Selected to represent sustained effort and persistence in pursuing longer-term objectives.
SC8	I often speak or act impulsively.	Reverse-coded	Impulsive action	Selected to represent a general tendency toward poorly inhibited verbal or behavioral responses.

Note. BSCS = Brief Self-Control Scale. The eight items were selected and, where necessary, contextually reworded from BSCS-related content to retain broad coverage of resistance to temptation, deliberate action, attentional control, behavioral inhibition, resistance to immediate gratification, concentration, goal-directed persistence, and impulsive action while reducing respondent burden. Items SC2, SC5, SC6, and SC8 were reverse coded. Previous evidence supporting shortened BSCS configurations among Chinese students is cited as general psychometric support for using a concise BSCS-based measure; it should not be interpreted as evidence that the exact item configuration used in the present study had previously undergone independent validation. In the present sample,

standardized factor loadings ranged from 0.621 to 0.826, Cronbach's α was 0.891, composite reliability was 0.893, and average variance extracted was 0.514.

Supplementary Table S7. Distributional diagnostics and outlier sensitivity analysis

Diagnostic or structural path	Full sample (N = 378)	Sensitivity sample (N = 374)	Difference or interpretation
**Distributional diagnostics**			
Diagnostic	Result	Sensitivity sample, (N=374)	Interpretation
Observed-indicator skewness range	−0.547 to 1.828	–	No severe univariate skewness
Observed-indicator kurtosis range	−0.938 to 3.538	–	No severe univariate kurtosis
CV construct-score skewness	1.280	–	Positively skewed, low-frequency distribution
Multivariate kurtosis	89.656	–	Departure from strict multivariate normality
Multivariate kurtosis CR	14.678	–	Departure from strict multivariate normality
Potential multivariate outliers	4 Cases	Temporarily excluded	Identified using Mahalanobis distance
**Model-fit sensitivity results**			
Index	Full sample, (N=378)	Sensitivity sample, (N=374)	Difference
χ^2/df	1.110	1.129	0.019
GFI	0.904	0.902	-0.002
AGFI	0.893	0.890	-0.003
IFI	0.990	0.988	-0.002
TLI	0.989	0.987	-0.002
CFI	0.990	0.988	-0.002
RMSEA	0.017	0.019	0.002
PCLOSE	1.000	1.000	No change
**Structural-path sensitivity results**			
Structural path	Full sample	Sensitivity sample	Absolute difference
CV ➡ Anxiety	0.497	0.481	0.016
Anxiety ➡ Insomnia	0.656	0.652	0.004
CV ➡ Self-control	-0.382	-0.369	0.013

Insomnia ➡ PAS	0.427	0.426	0.001
Self-control ➡ PAS	-0.291	-0.294	0.003
CV ➡ PAS	0.175	0.193	0.018

Note： The sensitivity analysis temporarily excluded four cases identified as potential multivariate outliers based on Mahalanobis distance. The same measurement and structural specifications were retained. All six structural paths remained statistically significant, and the 95% bias-corrected bootstrap confidence intervals remained entirely above or below zero, as appropriate. The largest absolute change in a standardized structural coefficient was 0.018. CV = cyberbullying victimization; PAS = perceived academic stress; CR = critical ratio.

职业院校学生网络欺凌受害经历、焦虑、失眠、自我控制与学业压力知觉问卷

Bilingual Questionnaire on Cyberbullying Victimization, Anxiety, Insomnia, Self-Control, and Perceived Academic Stress among Vocational College Students

亲爱的同学：您好！本问卷仅用于学术研究，采取匿名方式填写。请根据您的真实情况作答，答案无对错之分。整份问卷预计用时约 8–10 分钟。

Dear participant: This questionnaire is for academic research only and is completed anonymously. Please answer according to your actual situation. There are no right or wrong answers. The questionnaire takes approximately 8–10 minutes.

一、作答说明

1. Instructions

1. 请独立完成问卷，不与他人讨论。

1. Please complete the questionnaire independently without discussing it with others.

2. 请根据各部分题目说明的时间范围作答。

2. Please answer according to the time frame specified in each section.

3. 每题只选一个最符合您情况的选项，并在对应方框内打“√”。

3. For each item, choose the one option that best fits your situation and tick the corresponding box.

4. 如无特别说明，题目中的“网络”包括社交媒体、聊天软件、论坛、游戏平台及其他线上互动场景。

4. Unless otherwise specified, “online” includes social media, chat applications, forums, gaming platforms, and other online interaction settings.

二、基本信息

2. Demographic Information

**1. 性别 / Gender**	□ 男 Male　□ 女 Female　□ 其他 Other　□ 不愿透露 Prefer not to say
**2. 年龄 / Age**	________ 岁 / years old

**3. 年级/ Year of study**	□ 一年级 First year □ 二年级 Second year □ 三年级 Third year □ 其他 Other
**4. 专业类别 / Major category**	□ 工科 Engineering □ 商科 Business □ 教育 Education □ 医护 Medical/Nursing □ 艺术 Arts □ 其他 Other
**5. 平均每日上网时长 / Average daily internet use**	□ 1 小时以下 Less than 1 hour □ 1–3 小时 1–3 hours □ 3–5 小时 3–5 hours □ 5 小时以上 More than 5 hours
**6.平均每日社交媒体使用时长 / Average daily social media use**	□ 1 小时以下 Less than 1 hour □ 1–2 小时 1–2 hours □ 2–4 小时 2–4 hours □ 4 小时以上 More than 4 hours
**注意力检测题 1 / Attention-check Item 1**	为确认您已认真阅读作答说明，请在下方选择"×"。<br><br>To confirm that you are reading the instructions carefully, please select "×" below.<br><br>□ √ □ ×

三、网络欺凌受害经历

3. Cyberbullying Victimization

说明：本部分条目基于 ECIPQ 受害维度并结合本研究情境进行修订与整合，不视为某一原版量表的逐字照搬。

Note: This section is a revised and integrated research version adapted from the ECIPQ victimization dimension for the present study context, rather than a verbatim reproduction of a single original scale.

请回想过去两个月，您是否在网络平台、社交媒体、聊天软件或手机信息中遭遇过以下情况？请在最符合您的选项下打“√”。

Thinking about the past two months, have you experienced any of the following on online platforms, social media, chat apps, or mobile messages? Please tick the option that best reflects your experience.

选项说明 / Response options: 0=从未 Never；1=1–2 次 Once or twice；2=每月 1–2 次 Once or twice a month；3=约每周 1 次 About once a week；4=每周多次 More than once a week

题项 / Item	0	1	2	3	4
CV1. 有人在网上对我说侮辱性、刻薄或伤人的话。<br>*Someone said insulting, mean, or hurtful things to me online.*	☐	☐	☐	☐	☐
CV2. 有人在网络上散布关于我的谣言或不实信息。<br>*Someone spread rumors or false information about me online.*	☐	☐	☐	☐	☐
CV3. 有人通过网络消息威胁、恐吓或故意吓唬我。<br>*Someone threatened, intimidated, or tried to scare me through online messages.*	☐	☐	☐	☐	☐
CV4. 有人故意把我排除在群聊、线上活动或社交互动之外。<br>*Someone deliberately excluded me from group chats, online activities, or social interactions.*	☐	☐	☐	☐	☐
CV5. 有人未经允许公开、转发或泄露我的私人信息。<br>*Someone shared, forwarded, or disclosed my private information without permission.*	☐	☐	☐	☐	☐
CV6. 有人未经允许截图、转发或传播我的私人聊天内容。<br>*Someone screenshotted, forwarded, or spread my private conversations without permission.*	☐	☐	☐	☐	☐
CV7. 有人上传让我难堪、尴尬或受羞辱的照片或视频。<br>*Someone uploaded photos or videos that made me feel embarrassed or humiliated.*	☐	☐	☐	☐	☐
CV8. 有人故意修改、恶搞或 P 图我的照片/视频来取笑我。<br>*Someone edited, manipulated, or mocked my photos/videos to make fun of me.*	☐	☐	☐	☐	☐
CV9. 有人冒用我的身份、账号，或假装是我在网上发言。<br>*Someone impersonated me or pretended to be me online.*	☐	☐	☐	☐	☐
CV10. 有人反复向我发送骚扰性、攻击性或令人不安的信息。<br>*Someone repeatedly sent me harassing, aggressive, or disturbing messages.*	☐	☐	☐	☐	☐
CV11. 有人利用匿名账号或隐藏身份持续针对我。<br>*Someone repeatedly targeted me through anonymous or hidden accounts.*	☐	☐	☐	☐	☐

四、焦虑

4. Anxiety

请根据您在过去两周的实际情况，判断以下问题出现的频率。

Over the past two weeks, how often have you been bothered by the following problems?

选项说明 / Response options:

0=完全没有 Not at all；1=好几天 Several days；2=一半以上天数 More than half the days；3=几乎每天 Nearly every day

题项 / Item	0	1	2	3
ANX1. 感到紧张、焦虑或神经绷得很紧。<br>*Feeling nervous, anxious, or on edge.*	☐	☐	☐	☐
ANX2. 无法停止担忧，或难以控制担忧。<br>*Not being able to stop or control worrying.*	☐	☐	☐	☐
ANX3. 对不同事情担心过多。<br>*Worrying too much about different things.*	☐	☐	☐	☐
ANX4. 难以放松下来。<br>*Having trouble relaxing.*	☐	☐	☐	☐
ANX5. 坐立不安，难以安静待着。<br>*Being so restless that it is hard to sit still.*	☐	☐	☐	☐
ANX6. 容易烦躁或恼怒。<br>*Becoming easily annoyed or irritable.*	☐	☐	☐	☐
ANX7. 觉得好像有什么不好的事情要发生。<br>*Feeling as if something awful might happen.*	☐	☐	☐	☐

注意力检测题 2 / Attention-check Item 2

8 + 2 等于多少?

What is 8 + 2?

五、失眠

5. Insomnia

请根据您在过去两周的睡眠情况作答。以下 1–3 题、4 题、5–7 题的选项含义不同，请分别查看各部分的选项说明后再勾选。

Please answer based on your sleep during the past two weeks. The response options differ for Items 1–3, Item 4, and Items 5–7. Please read each part’ s response guide carefully before ticking.

选项说明 / Response options: 请按各小节提示作答 / Please follow the response guide for each subsection.

（一）睡眠困难程度 / Part A. Severity of sleep difficulties

选项说明 / Response options: 0=没有问题 No problem；1=轻度 Mild；2=中度 Moderate；3=较重 Severe；4=非常严重 Very severe

题项 / Item	0	1	2	3	4
INS1. 入睡困难的程度。<br>*Difficulty falling asleep.*	☐	☐	☐	☐	☐
INS2. 夜间易醒或醒后难再入睡的程度。<br>*Difficulty staying asleep or returning to sleep after waking.*	☐	☐	☐	☐	☐
INS3. 过早醒来的程度。<br>*Problem with waking up too early.*	☐	☐	☐	☐	☐

（二）睡眠满意度 / Part B. Satisfaction with sleep

选项说明 / Response options: 0=非常满意 Very satisfied；1=比较满意 Satisfied；2=一般 Neutral；3=不太满意 Dissatisfied；4=非常不满意 Very dissatisfied

题项 / Item	0	1	2	3	4
INS4. 你对自己目前睡眠状况的满意程度。<br>*How satisfied are you with your current sleep pattern.*	☐	☐	☐	☐	☐

（三）睡眠问题的影响与困扰 / Part C. Impact and distress

选项说明 / Response options: 0=完全没有 Not at all；1=轻微 A little；2=中等 Somewhat；3=较多 Much；4=非常多 Very much

题项 / Item	0	1	2	3	4
INS5. 睡眠问题对你白天学习、生活或功能状态的影响程度。<br>*To what extent do your sleep problems interfere with your daytime functioning?*	☐	☐	☐	☐	☐
INS6. 你的睡眠问题在别人眼中有多明显。<br>*How noticeable do you think your sleep problem is to others?*	☐	☐	☐	☐	☐
INS7. 你对当前睡眠问题的担忧或苦恼程度。<br>*How worried or distressed are you about your current sleep problem?*	☐	☐	☐	☐	☐

六、自我控制

6. Self-Control (Abbreviated BSCS-Based Research Version)

为控制整份问卷长度并降低后半段作答疲劳，本部分采用基于 BSCS 精简整合形成的 8 题研究版条目。以下题目反映您平时的行为习惯和自我管理方式，请根据您的总体情况作答。

To reduce overall questionnaire length and minimize respondent fatigue, this section uses an 8-item abbreviated research version adapted from the BSCS. The following items reflect your general behavioral habits and self-management style. Please answer based on your overall situation.

选项说明 / Response options: 1=非常不符合 Not at all like me；2=比较不符合 Slightly unlike me；3=一般 Neutral；4=比较符合 Mostly like me；5=非常符合 Very much like me

题项 / Item	1	2	3	4	5
SC1. 我能较好地抵制诱惑。<br>*I am good at resisting temptation.*	☐	☐	☐	☐	☐
SC2. 我常常还没想清楚就行动。<br>*I often act before thinking things through.*	☐	☐	☐	☐	☐
SC3. 我能专注于真正重要的事情。<br>*I am able to stay focused on what is important.*	☐	☐	☐	☐	☐
SC4. 当某件事不合适时，我能控制自己不去做。<br>*I can stop myself from doing something inappropriate.*	☐	☐	☐	☐	☐
SC5. 娱乐和享乐有时会妨碍我完成该做的事情。<br>*Fun and enjoyment sometimes keep me from getting my work done.*	☐	☐	☐	☐	☐
SC6. 我有时很难集中注意力去做该做的事。<br>*I sometimes have difficulty concentrating on what I should do.*	☐	☐	☐	☐	☐
SC7. 我能持续朝长期目标努力。<br>*I am able to work steadily toward long-term goals.*	☐	☐	☐	☐	☐
SC8. 我经常冲动地说话或做事。<br>*I often speak or act impulsively.*	☐	☐	☐	☐	☐

七、学业压力知觉

7. Perceived Academic Stress (Task- and Workload-Oriented Items)

请根据您在本学期以来的整体学业体验，判断以下陈述与您的符合程度。

Please indicate how well the following statements describe your academic experience during this semester.

选项说明 / Response options: 1=非常不同意 Strongly disagree；2=不同意 Disagree；3=一般 Neutral；4=同意 Agree；5=非常同意 Strongly agree

题项 / Item	1	2	3	4	5
AS1. 我觉得自己目前要完成的学习任务超过了我可以轻松应对的范围。 / I feel that my current academic tasks are more than I can comfortably handle.	□	□	□	□	□
AS2. 考试、测验或考核常常给我带来较大的学习压力。 / Exams, tests, or assessments often place considerable academic pressure on me.	□	□	□	□	□
AS3. 我觉得自己没有足够的时间完成作业、课程任务或实践训练。 / I feel that I do not have enough time to complete assignments, coursework, or practical training tasks.	□	□	□	□	□
AS4. 我经常感受到来自成绩、排名或表现要求的压力。 / I often feel pressure related to grades, ranking, or performance requirements.	□	□	□	□	□
AS5. 我觉得老师、家长或他人对我的学习表现期望较高。 / I feel that teachers, parents, or others have high expectations for my academic performance.	□	□	□	□	□

AS6. 学业任务占用了我大量休息和放松的时间。 / Academic tasks take up a great deal of my time for rest and relaxation.	□	□	□	□	□
AS7. 对我来说，平衡理论课程、技能训练、实习或实训任务是有压力的。 / Balancing theory courses, skills training, internships, or practicum tasks is stressful for me.	□	□	□	□	□
AS8. 不同课程或训练任务的截止时间经常集中出现，让我感到学业负担较重。 / Deadlines from different courses or training tasks often cluster together, making my academic workload feel heavy.	□	□	□	□	□

感谢您的参与和支持！如果您在作答过程中感到明显不适，或近期持续存在焦虑、睡眠困扰和学习压力，建议及时联系学校心理中心、辅导员或专业心理/医疗服务人员。

Thank you for your participation and support. If you felt notably distressed while completing this questionnaire, or if you have been experiencing persistent anxiety, sleep problems, or academic stress, please consider contacting your campus counseling center, advisor, or a mental health/medical professional.

Note. The two attention-check items were used solely for response-quality screening and were not included in the scoring of any substantive study construct. A response was excluded if either attention-check item was answered incorrectly or if one or more substantive questionnaire items were left unanswered.
